# Astrocytic RNA editing regulates the host immune response to alpha-synuclein

**DOI:** 10.1126/sciadv.adp8504

**Published:** 2025-04-11

**Authors:** Karishma D'Sa, Minee L. Choi, Aaron Z. Wagen, Núria Setó-Salvia, Olga Kopach, James R. Evans, Margarida Rodrigues, Patricia Lopez-Garcia, Joanne Lachica, Benjamin E. Clarke, Jaijeet Singh, Ali Ghareeb, James Bayne, Melissa Grant-Peters, Sonia Garcia-Ruiz, Zhongbo Chen, Samuel Rodriques, Dilan Athauda, Emil K. Gustavsson, Sarah A. Gagliano Taliun, Christina Toomey, Regina H. Reynolds, George Young, Stephanie Strohbuecker, Thomas Warner, Dmitri A. Rusakov, Rickie Patani, Clare Bryant, David A. Klenerman, Sonia Gandhi, Mina Ryten

**Affiliations:** ^1^ Department of Clinical and Movement Neurosciences, UCL Queen Square Institute of Neurology, Queen Square, London WC1N 3BG, UK.; ^2^ The Francis Crick Institute, 1 Midland Road, London NW1 1AT, UK.; ^3^ Aligning Science Across Parkinson’s (ASAP) Collaborative Research Network, Chevy Chase, MD 20815, USA.; ^4^ Department of Brain & Cognitive Sciences, KAIST, 921 Dehak-ro, Daejeon, Republic of Korea.; ^5^ Department of Genetics and Genomic Medicine, Great Ormond Street Institute of Child Health, University College London, London WC1N 1EH, UK.; ^6^ Reta Lila Weston Institute, UCL Queen Square Institute of Neurology, London WC1N 3BG, UK.; ^7^ Department of Clinical and Experimental Epilepsy, UCL Queen Square Institute of Neurology, London WC1N 3BG, UK.; ^8^ Neuroscience and Cell Biology Research Institute, City St George’s, University of London, Cranmer Terrace, London SW17 0RE, UK.; ^9^ Department of Chemistry, University of Cambridge, Cambridge CB2 1EW, UK.; ^10^ UK Dementia Research Institute at The University of Cambridge, Cambridge CB2 0AH, UK.; ^11^ UCL Queen Square Institute of Neurology, University College London, London WC1N 3BG, UK.; ^12^ Department of Neuromuscular Disease, UCL Queen Square Institute of Neurology, Queen Square, London WC1N 3BG, UK.; ^13^ Applied Biotechnology Lab, The Francis Crick Institute, London NW1 1AT, UK.; ^14^ Nuffield Department of Orthopaedics, Rheumatology, and Musculoskeletal Sciences, University of Oxford, Oxford OX3 7LD, UK.; ^15^ FutureHouse, 1405 Minnesota Street, San Francisco, CA 94107, USA.; ^16^ Montréal Heart Institute, Montréal, QC, Canada.; ^17^ Department of Medicine and Department of Neurosciences, Université de Montréal, Montréal, QC, Canada.; ^18^ MRC Laboratory of Medical Sciences, London W12 0HS, UK.; ^19^ Department of Veterinary Medicine, University of Cambridge, Cambridge CB3 0ES, UK.; ^20^ Department of Clinical Neurosciences, School of Clinical Medicine, University of Cambridge, Cambridge CB2 0SP, UK.; ^21^ Department of Genetics, University of Cambridge, Cambridge CB2 3EH, UK.

## Abstract

RNA editing is a posttranscriptional mechanism that targets changes in RNA transcripts to modulate innate immune responses. We report the role of astrocyte-specific, ADAR1-mediated RNA editing in neuroinflammation in Parkinson’s disease (PD). We generated human induced pluripotent stem cell–derived astrocytes, neurons and cocultures and exposed them to small soluble alpha-synuclein aggregates. Oligomeric alpha-synuclein triggered an inflammatory glial state associated with Toll-like receptor activation, viral responses, and cytokine secretion. This reactive state resulted in loss of neurosupportive functions and the induction of neuronal toxicity. Notably, interferon response pathways were activated leading to up-regulation and isoform switching of the RNA deaminase enzyme, ADAR1. ADAR1 mediates A-to-I RNA editing, and increases in RNA editing were observed in inflammatory pathways in cells, as well as in postmortem human PD brain. Aberrant, or dysregulated, ADAR1 responses and RNA editing may lead to sustained inflammatory reactive states in astrocytes triggered by alpha-synuclein aggregation, and this may drive the neuroinflammatory cascade in Parkinson’s.

## Teaser

RNA editing and astrocytic inflammatory response pathways may be therapeutic targets for the treatment of Parkinson’s disease.

## INTRODUCTION

Parkinson’s disease (PD) is a progressive neurodegenerative condition characterized by the accumulation of intraneuronal aggregates of alpha-synuclein (α-syn) through the brain (*1*). Astrogliosis has been reported in postmortem Parkinson’s brain (*2*–*7*) and in rodent models of synucleinopathy (*8*, *9*), and astrocytes accumulate α-syn inclusions (*10*), raising a role for astrocytes and astrocyte-mediated immune cascades (*11*) in triggering or driving PD pathogenesis. Astrocytes are an abundant glial cell essential for neuronal function and survival through the maintenance of central nervous system (CNS) homeostasis via modulation of neurotransmitters and synapse formation, ionic balance, and maintenance of the blood-brain barrier (*12*). Reactive astrogliosis defines a process whereby, in response to pathology, astrocytes undergo changes in transcriptional regulation and biochemical, morphological, metabolic, and physiological remodeling, which ultimately result in a switch from resting to reactive states. The nature of the underlying stimulus (neuroinflammatory versus ischemic) has defined certain reactive astrocyte states into neurotoxic (so-called A1, which promote lipid mediated neuronal death via activated microglia) and neuroprotective (so-called A2, which promote neuronal survival and regeneration through neurotrophic factors) (*13*). Reactive transformation can be associated with loss of neurosupportive and homeostatic functions, reduced synapse formation (*14*), alterations in glutamate uptake and recycling (*15*), and dysregulated calcium signaling (*16*). Simultaneously reactive states are associated with the gain of neurotoxic properties (*17*).

Focusing on PD, there is evidence that A1 astrocytes have been shown to infiltrate brain regions associated with PD, including the striatum (*13*). Astrocytes in these regions express the highest regional levels of immune mediators, such as toll-like receptor 4 (TLR4) and myeloid differentiation primary response 88 (MyD88), correlating with PD pathology (*18*). Of note, inhibiting the formation of these A1 astrocytes by blocking microglia-astrocytic cross-talk is neuroprotective in mouse models of PD (*19*). Reactive astrocyte substates are likely to be diverse beyond these two states (*14*, *20*) and involve several exogenous and endogenous triggers such as damage-associated molecular patterns (DAMPs) or proteostatic stresses induced by misfolded proteins. Compared to control cell lines, human astrocytic models of PD release increased amounts of proinflammatory cytokines in response to α-syn (*21*). Moreover, in response to α-syn fibrils, induced pluripotent stem cell (iPSC)–derived astrocytes can assume an antigen-presenting function, with up-regulation of major histocompatibility complex genes and relocation of human leukocyte antigen molecules to the cell surface to present α-syn fibril peptides to neighboring cells (*21*). This finding, replicated in postmortem brain samples (*22*), supports the idea that astrocytes may mediate the immune response in the brain.

We have recently reported that physiological concentrations of oligomeric α-syn trigger a TLR4-dependent inflammatory response in murine primary astrocytes (*18*). The small soluble hydrophobic and β sheet–rich aggregates or oligomers of α-syn are known to be neurotoxic and trigger cell-selective processes that are specific to the structural conformation of the protein (*23*–*26*).

Substate modeling has been achieved in human iPSC (hiPSC)–derived astrocytes and cocultures (*27*), which enable capture at a molecular level of cell autonomous and non–cell autonomous cascades in pathology. We investigate how hiPSC-derived astrocytes respond to α-syn oligomers (αsyn-O), which downstream response pathways are activated, and how this reactivity affects neurons. We integrate bulk and single-cell transcriptomic, functional, and biophysical approaches in five lines of hiPSC-derived astrocytes (three in-house and two commercial lines), both alone and in coculture with neurons, to define the molecular response of astrocytes to misfolded α-syn. Last, identifying innate immune pathways of interest, we explore those pathways in postmortem brain samples.

## RESULTS

### Generation and functional characterization of hiPSC-derived astrocytes and neurons alone or in coculture

**Fig. 1.**
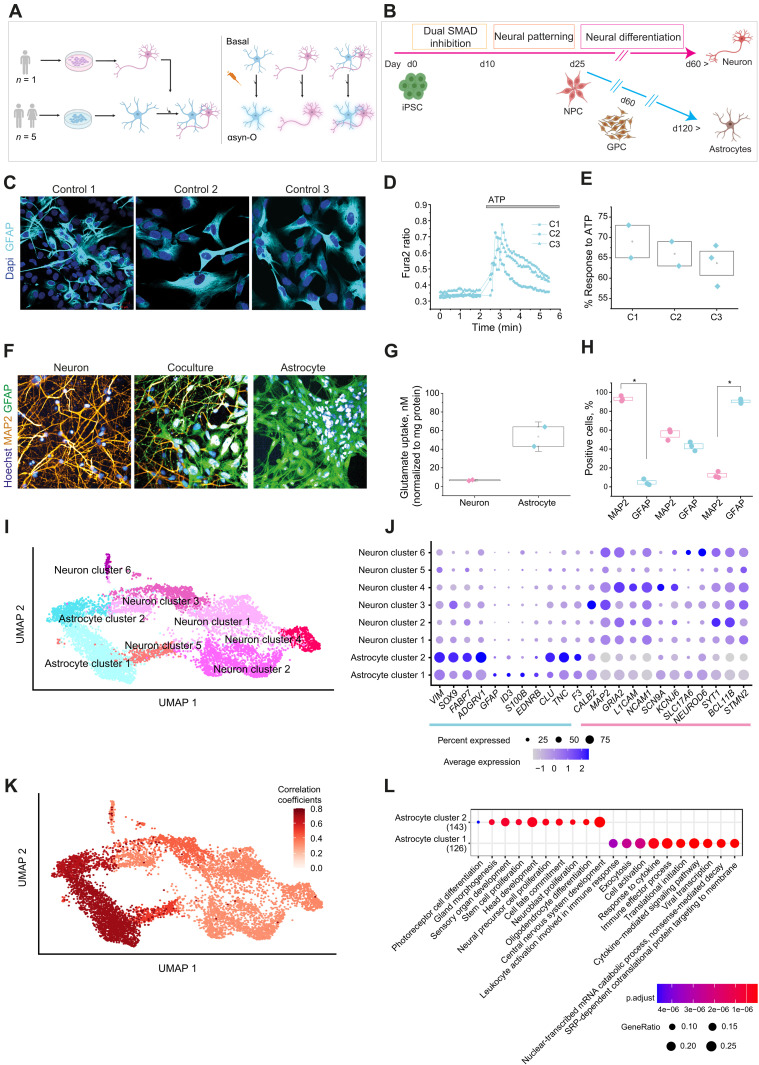
Schematic illustrations of experimental paradigm and the differentiation protocols. (**A**) hiPSC-derived from five healthy individuals are differentiated into astrocytes (in-house lines: C1, C2, and C3; commercial lines from ACS: C4 and C5) and from one individual into neurons. (**B**) Differentiation of cortical region–specific astrocytes was performed, modifying established protocols (*78*, *79*). NPCs using an established protocol [Shi *et al.*, 2012 (*29*)] were differentiated into either neurons or GPC (glial precursor cells) after 30 days from NPC and then further differentiated into mature astrocytes. (**C**) Immunocytochemistry images show that three lines of hiPSC-derived astrocytes express the astrocytic marker, GFAP. (**D** and **E**) There is a calcium response to ATP in iPSC-derived astrocytes. Representative traces of calcium (D) and the percentage of cells (E) in response to ATP. (**F**) hiPSC-derived astrocyte enables uptake of glutamate (Glutamate Assay Kit, ab83389/K629-100, Abcam). (**G** and **H**) Composition of hiPSC-derived neuron and astrocyte coculture assessed using MAP2 (neuronal marker) and GFAP (astrocytic marker) immunocytochemistry together with representative images of a neuronal, astrocyte, and coculture (G) and the quantification (H). (**I**) Uniform Manifold Approximation and Projection (UMAP) plot showing the clustering of the integrated dataset using the cells from all the samples (basal and αsyn-O–treated astrocytes, neurons, and coculture samples). (**J**) Dot plots showing the expression of the astrocyte and neuron marker genes in the clusters identified in the single-cell RNA-seq data. (**K**) UMAP overlaid with the correlation coefficients, showing the correlation of the two astrocyte clusters with the astrocytes from Leng *et al.* (*27*). (**L**) Gene Ontology (GO) terms associated with the genes up-regulated in astrocyte clusters 1 and 2. (A) and (B) were created with BioRender (https://biorender.com/)

We generated hiPSC-derived astrocytes from five healthy donors and cortical neurons from a sixth healthy donor (table S1) (*28*). We compared the cellular response to αsyn-O in astrocyte-only, neuron-only, and astro-neuronal cultures (Fig. 1A). Highly enriched cultures of cortical astrocytes in feeder-free conditions were generated from three in-house lines using an optimized small-molecule serum-free protocol, and two were purchased commercially, derived through a proprietary serum-free protocol (Fig. 1B). We used the Shi *et al.* (*29*) protocol (Fig. 1B) to generate highly enriched (>90%) and functional hiPSC-derived cortical neurons from neural precursor cells (NPCs) (*24*, *25*).

Immunolabeling with glial fibrillary acidic protein (GFAP), an astrocyte-specific marker, demonstrated that cultures contained >90% mature astrocytes (Fig. 1C). hiPSC-derived astrocytes also displayed cytosolic calcium responses on application of adenosine triphosphate (ATP) as assessed using Fura-2 (Fig. 1, D and E) (*30*). All lines demonstrated appropriate Na^+^-dependent uptake of glutamate from the extracellular space, one of the major astrocytic functions that indicate functional activity of excitatory amino acid transporter 1/2 in hiPSC-derived astrocytes (Fig. 1G). Together, the ATP-dependent calcium responses and glutamate uptake confirmed generation of functionally mature astrocytes.

To investigate how astrocytes affect neuronal function, we generated cocultures by plating hiPSC-derived cortical astrocytes and neurons in a 1:1 ratio and cultured for at least 3 days before use. The composition of cocultures was assessed by immunocytochemistry using both MAP2, a neuronal marker, and GFAP, an astrocytic marker (Fig. 1, F and H). The cocultures contained 55.9 ± 3.4% MAP2-positive cells and 42.9 ± 2.7% GFAP-positive cells, while neuronal cultures contained 93.2 ± 1.7% MAP2-positive cells and astrocytic cultures contained 90.5 ± 1.3% GFAP-positive cells.

To generate oligomers of α-syn, human recombinant monomeric wild-type (WT) α-syn was aggregated in the dark at 37°C and 200 rpm, for ~7 to 8 hours. We took aliquots of α-syn solution directly from an aggregation reaction at different time points, and we have characterized them using two single-molecule methods: single-molecule fluorescence resonance energy transfer (FRET) efficiency (of labeled recombinant α-Syn) and single-molecule aggregate visualization by enhancement (of unlabeled recombinant α-Syn, which uses single-molecule fluorescence microscopy to detect the benzothiazole salt Thioflavin-T (ThT). Upon binding to β sheet structures, ThT fluorescence increases, allowing individual aggregated species to be detected. At the time point selected for these experiments (end of the lag phase), the preparation is 1% oligomeric, and the rest is monomer. These aggregates are smaller than 20-mers, soluble, and have β sheet conformation, and application of these aggregates to glial cells previously caused tumor necrosis factor–α (TNF-α) production via TLR4 in cells, while there was no signaling from the equivalent monomeric protein (*18*, *24*, *25*, *31*, *32*).

Whole-cell patch-clamp recording of neurons was performed in neuron-only and astro-neuronal cultures to assess the electrophysiological properties and excitability of the cells. In neuron-only cultures, neurons (~100 days in vitro) displayed a relatively depolarized resting membrane potential (*V*rest) compared with age-matched cells in cocultures (−45.9 ± 2.8 mV, *n* = 33 versus –56.0 ± 2.1 mV, *n* = 34, *P* = 0.0053, respectively; fig. S1, A and B). No significant difference was observed in neuronal capacitance across culture types (*C*_m_: 44.8 ± 3.5 pF, *n* = 35 in cultures and 43.7 ± 3.8 pF, *n* = 40 in cocultures, *P* = 0.843; (fig. S1C). Input resistance (fig. S1D) and the time constant (fig. S1E) were significantly altered in astro-neuronal cultures. In neuron-only cultures, neurons generated a single action potential (AP) in response to current injection, whereas neurons in astro-neuronal cultures generated a train of induced APs in either step-wise depolarizing protocol or slow-injecting ramp current (fig. S1F). The parameters of AP spike (threshold, spike amplitude, and kinetics) also confirmed that cocultures altered the neuronal performance. Neurons were more excitable in cocultures, as a lesser current was required to bring neurons to drive firing (fig. S1, G to J).

### Single-cell RNA-seq of astrocyte-only, neuron-only, and astro-neuronal cultures

We used RNA sequencing (RNA-seq) to further characterize hiPSC-derived astrocytes and neurons. After 120 days of differentiation, all cells were harvested with and without αsyn-O stimulation, each with a technical replicate resulting in 44 samples. These samples underwent bulk RNA-seq, and six samples were also sequenced with single-cell technology (astrocyte-only, neuron-only, and astro-neuronal cultures, with and without αsyn-O). Across the integrated single-cell dataset, we identified eight cell clusters, which include both astrocytic and neuronal subtypes. Cell types were assigned using a set of previously published and curated marker genes (*33*, *34*). On the basis of the expression of these marker genes, we identified two astrocytic and six neuronal clusters (Fig. 1, I and J). Given that hiPSC-derived astrocytic profiles have been previously well characterized by Leng and colleagues (*27*), we initially assessed our clusters for correlations in gene expression globally with the reported iAstrocytes transcriptomic profiles and found that they were highly correlated with both of our astrocyte clusters (*r* = 0.71 and 0.64) (Fig. 1K), subsequently referred to as astrocyte cluster 1 (AC1) and AC2.

Given the growing literature on astrocytic subtypes, we investigated the clusters further. Focusing specifically on genes differentially expressed between the clusters (AC1 and AC2), we identified 300 genes of interest [at false discovery rate (FDR) <5% and >twofold change in expression] of which 129 were more highly expressed in AC1 and 171 genes which were more highly expressed in AC2 (table S2). Gene set enrichment analysis of these differentially expressed genes identified immune and cytokine-related terms among genes more highly expressed in AC1. Conversely, genes more highly expressed in AC2 showed enrichment for terms related to morphogenesis, regulation of development, and differentiation (Fig. 1L and table S3). Furthermore, we noted that the pattern of gene expression in the AC2 cluster appeared to resemble that described for neuroprotective astrocytes (fig. S2A). Thus, we identified two subtypes of astrocytes, AC1 and AC2, which differed in terms of their inflammatory and protective transcriptomic profiles.

### hiPSC-derived astrocytes are reactive to αsyn-O and secrete cytokines

**Fig. 2.**
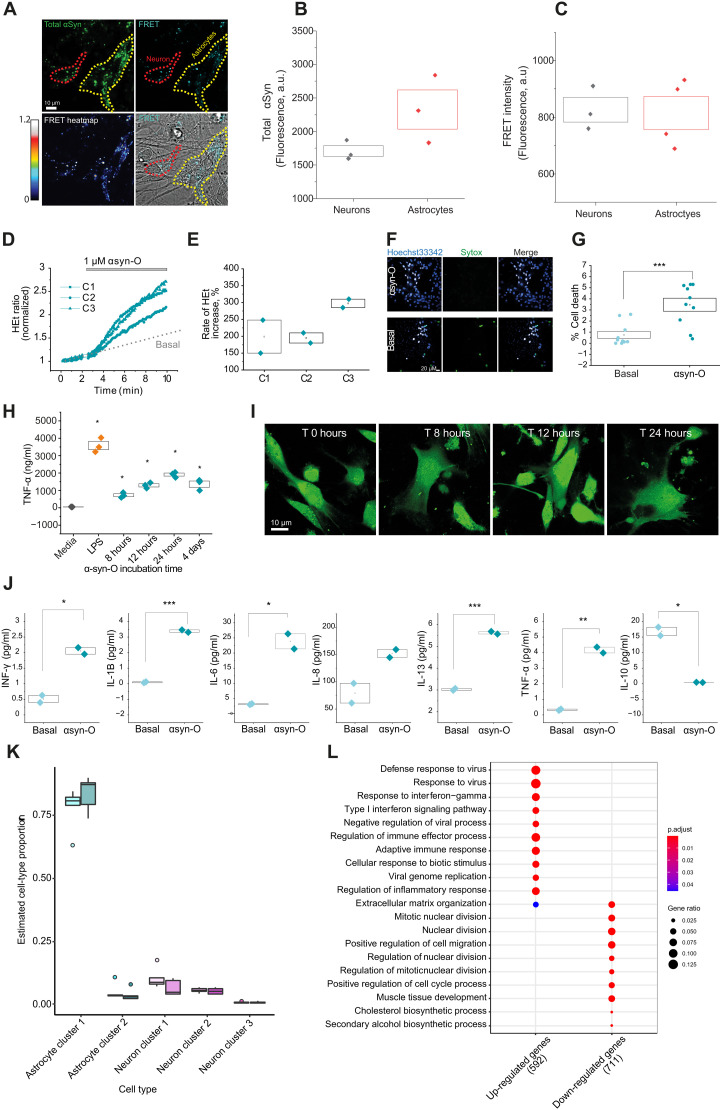
Oligomer treatment of astrocytes induces an inflammatory state. (**A**) Uptake of monomeric species of labeled α-syn (A53T monomer) in astrocytes and neurons (yellow and red dotted lines, respectively) was detected, and formation of oligomers inside cells confirmed using FRET. (**B** and **C**) No difference in the formation of oligomeric species observed despite higher uptake of total α-syn in astrocytes compared to neurons (*n* = 3 independent inductions). (**D** and **E**) Application of αsyn-O induces overproduction of ROS. (**F** and **G**) Cell death induced by αsyn-O detected in astrocytes at low levels. (**H**) Cytokine release in αsyn-O–treated astrocytes is time dependent with TNF-α level highest after 24 hours of incubation with αsyn-O. (**I**) Morphological changes were traced using Fluo4 across the same time course as the cytokine release. (**J**) αsyn-O–treated astrocytes induce an inflammatory state by releasing a range of cytokines (basal, αsyn-O treated for interleukin-13 (IL-13), 3.02 ± 0.04 and 5.62 ± 0.05 pg/ml; IL-6, 3.11 ± 0.13 and 23.8 ± 2.4 pg/ml; IL-8, 79.11 ± 18.07 and 152.01 ± 7.29 pg/ml; IL-1B, 0.955 ± 0.03011 and 3.38 ± 0.0772; INF-γ, 0.51 ± 0.11 and 2.05 ± 0.1 pg/ml; TNF-α, 0.33 ± 0.0414 and 4.16 ± 0.187). (**K**) Cell-type proportions (excluding cell types with proportions <1%) for each of the cell types in the bulk astrocyte samples, basal, and αsyn-O treated, predicted by Scaden. (**L**) Top 10 GO terms associated with up- and down-regulated differentially expressed genes at FDR < 5% and ≥ twofold change in expression in the astrocytes basally versus with αsyn-O treatment. a.u., arbitrary unit.*P < 0.05, **P < 0.01, ***P < 0.001.

We treated astrocytes and neurons alone, and in coculture, with fluorescently labeled AF488-WT α-syn monomers at 500 nM and imaged the intracellular fluorescence for over 12 hours. Both astrocytes and neurons alone, and in coculture, can internalize exogenously applied α-syn (fig. S3A) within 12 hours. We examined whether hiPSC-derived astrocytes can induce the formation of αsyn-O using a FRET biosensor, which enables visualization of oligomers in cells (*25*). We treated hiPSC-derived astrocytes with two populations of fluorescently tagged α-syn (AF488- and AF594-tagged A53T α-syn monomers, total of 500 nM) for 24 hours and then measured the intracellular accumulation of α-syn (“total α-syn”) based on the intensity of AF594 through direct excitation with 594 nm irradiation. The formation of oligomer (“FRET”) was visualized via the presence of signal from the acceptor fluorophore (AF594) after excitation of the donor fluorophore (488-nm irradiation) (Fig. 2A). Using this approach, we found that total α-syn uptake was higher in hiPSC-derived astrocytes than in neurons, while similar levels of de novo oligomers (FRET) were detected in both cell preparations (Fig. 2, B and C).

αsyn-O exposure can cause cell toxicity and excessive reactive oxygen species (ROS) generation in primary astrocytes (*18*). To determine whether hiPSC-derived astrocytes show similar responses, we assessed ROS production using dihydroethidium (HEt) dye, which allowed us to robustly measure the rate of oxidation of the dye by cellular superoxide production (*35*). We found that ROS production significantly increased compared to basal levels (normalized to 100%; Fig. 2, D and E) in hiPSC-derived astrocytes after the application of αsyn-O. This was associated with a very low level of cell death (Fig. 2, F and G).

Since our previous study showed that αsyn-O treatment triggers an inflammatory response in primary astrocytes with associated increases in cytokine release (*18*), we assessed this phenomenon in hiPSC-derived astrocytes. In agreement with our previous findings, recombinant α-syn monomer and recombinant β-synuclein monomer did not induce an inflammatory response in astrocytes (fig. S3B). We tested a range of αsyn-O concentrations (100 nM to 2 μM) and a range of incubation times (8 hours to 4 days), collected media, and measured TNF-α release (Fig. 2H and fig. S3C).

On the basis of these data, we selected a 24-hour incubation time point, at a concentration of 1 μM αsyn-O (10 nM oligomer) and assessed the cytokine profiles in the media using an MSD (V-PLEX Proinflammatory Panel 1) electrochemiluminescence assay kit. We found that αsyn-O treatment of hiPSC-derived astrocytes consistently induced a significant increase in the secretion of a variety of cytokines compared to media-only cultures. Thus, we demonstrated that αsyn-O exposure induces hiPSC-derived astrocytes to become proinflammatory (Fig. 2, H to J).

### Oligomer treatment of hiPSC-derived astrocytes triggers antiviral inflammatory responses

Next, we used bulk RNA-seq to investigate hiPSC-derived astrocyte responses to αsyn-O treatment in more detail. Given that we identified two major astrocytic clusters (AC1 and AC2), we determined whether αsyn-O changes the relative proportions of these cell clusters. With this in mind, we used the tool Scaden (*36*) together with our single-cell RNA-seq data (Materials and Methods) to estimate cluster proportions in bulk RNA-seq data across the whole dataset, noting a high correlation in cell-type proportion estimates based on single-cell and Scaden-derived data (fig. S2B). This approach was based on the colocalization of all astrocytic samples within exploratory principal components analyses based on bulk RNA-seq data, suggesting that the astrocyte clusters we identified in a subset of samples were present in all. While we found no significant change in astrocyte subtype proportion between basal and αsyn-O–treated astrocyte cultures (Fig. 2K and table S4), significant changes in gene expression and splicing were observed.

We identified 2004 genes that were significantly differentially expressed (8.17 at FDR <5% and at least twofold change in expression) following treatment of hiPSC-derived astrocytes with αsyn-O, of which 917 were up-regulated and 1087 were down-regulated in the treated astrocytes (table S5). These up-regulated genes with at least twofold change in expression were enriched for those implicated in viral responses, including “defense response to virus,” “response to interferon-gamma,” and “type I interferon signaling pathway” (Fig. 2L and table S6). These results were highly consistent with the functional data demonstrating a robust cytokine response to αsyn-O treatment. Since splicing analyses have been shown to provide distinct biological information (*37*–*39*), this form of analysis was used to further characterize hiPSC-derived astrocyte responses to αsyn-O. We identified 707 significant differentially spliced intron clusters corresponding to 590 genes (FDR < 0.05, |∆PSI| ≥ 0.1) with significant enrichment for cytoskeletal terms, potentially reflecting the observed morphological changes in astrocytes with oligomer treatment (fig. S3, D to F, and tables S7 to S9). Morphological changes induced by αsyn-O are evaluated through astrocyte segmentation, followed by quantification of GFAP pixel area and intensity based on the distance from the nuclear membrane. This approach assesses both morphological polarity and intensity gradients (*40*, *41*), providing a detailed understanding of the spatial distribution and intensity changes in astrocytic morphology thus enabling the tracking of reactive astrocytic morphology upon αsyn-O stimulation.

### hiPSC-derived astrocytes maintain inflammatory states on exposure to αsyn-O in coculture

**Fig. 3.**
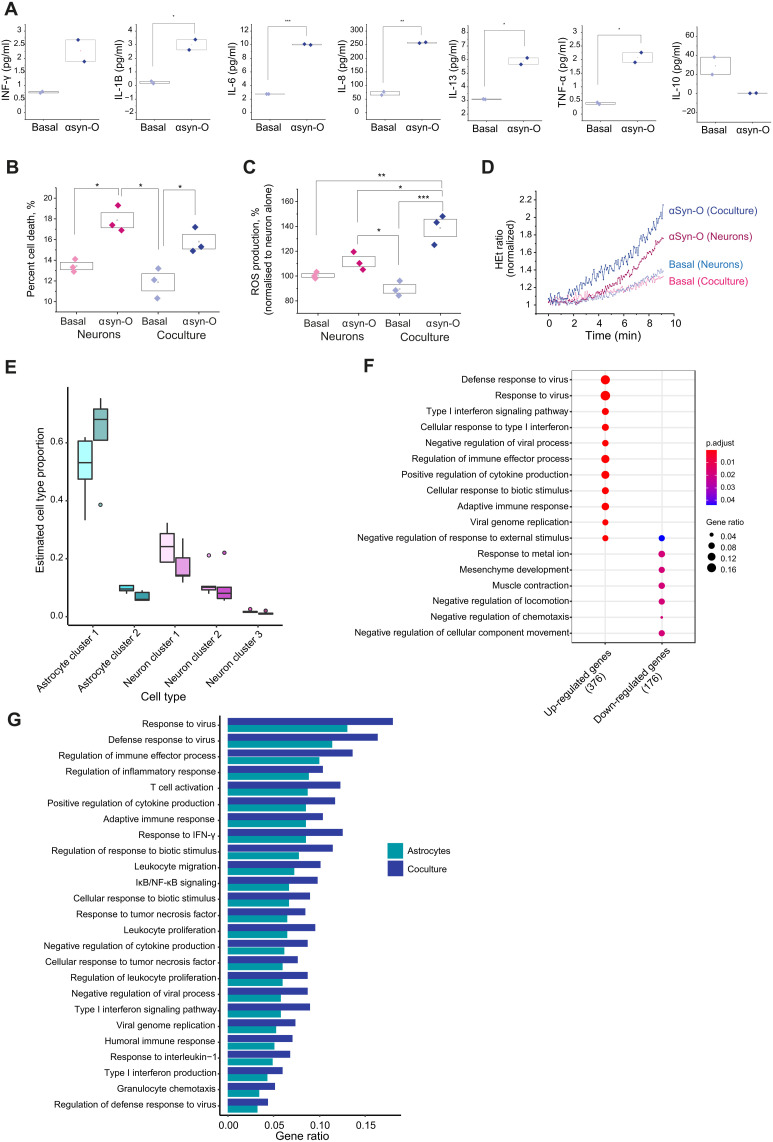
Coculture setting provides evidence of the astrocytes becoming more inflammatory. (**A**) Effect of αsyn-O on cytokine release in the cocultures (basal IL-13: 3.091 ± 0.0139 pg/ml and αsyn-O treated 5.88 ± 0.238 pg/ml; IL-6: basal 2.75 ± 0.00174 pg/ml, αsyn-O treated 10.026 ± 0.0593 pg/ml; IL-8: basal 71.09 ± 6.107 pg/ml, αsyn-O treated 257.2 ± 1.58 pg/ml; IL-1B: basal: 0.231 ± 0.0835 pg/ml, αsyn-O treated 2.99 ± 0.381 pg/ml; INF-γ: basal 0.392 ± 0.0329, αsyn-O treated 2.078 ± 0.180 pg/ml; TNF-α: basal 0.392 ± 0.0329, αsyn-O treated 2.078 ± 0.1801) (**B** to **D**) αsyn-O induces toxicity in neurons alone and in coculture with astrocytes and induces increased levels of ROS in neurons alone and in coculture. (**E**) Plot showing the cell-type proportions estimated by Scaden from the single-cell data in the coculture samples, basally (lighter shade) and with αsyn-O treatment (darker shade). There is a significant difference between the cell-type proportions of astrocyte cluster 1 and 2 in the coculture with αsyn-O treated compared to basal. (**F**) GO terms associated with the up- and down-regulated differentially expressed genes with at least ≥twofold change in expression in the coculture αsyn-O treated versus basal. (**G**) Visualizing gene ratios of the enrichments observed in the up-regulated differentially expressed genes of the astrocytes (cyan) compared to that of the coculture (blue) (*P < 0.05, **P < 0.01, ***P < 0.001).

First, we studied the functional effects of αsyn-O treatment on astro-neuronal cultures. Media collected from the samples were used to measure a range of cytokines secreted from the cells using an MSD (V-PLEX Proinflammatory Panel 1) electrochemiluminescence assay kit. αsyn-O induced more secretion of a variety of cytokines compared to untreated cocultures demonstrating inflammatory activation of the coculture (Fig. 3A).

Previously, we have demonstrated that αsyn-O induced an increased level of neuronal death and oxidative stress (*18*, *24*, *25*). Using live-cell imaging, we showed that activated astro-neuronal cultures were associated with higher levels of cell death (Fig. 3B) and higher levels of ROS production (Fig. 3, C and D). Furthermore, patch-clamp recordings performed in cocultures treated with αsyn-O demonstrated a drop in the *V*rest after treatment (fig. S4B) and increased input resistance (fig. S4C) in neurons. Neurons also had impaired firing, with a dramatically changed AP waveform. The threshold for AP spike generation was depolarized (fig. S4, D and E), the amplitude was reduced (fig. S4F), and the spike was significantly extended compared with control astro-neuronal cultures. Thus, the proteinopathy appeared to induce an activated inflammatory state of astrocytes, which is associated with loss of the previous neuronal supportive function seen with resting astrocytes. In addition, there are toxic gain-of-function effects in both astrocytes and neurons in coculture, such as induction of oxidative stress, altered excitability, and neuronal cell death.

As before, we also combined single-cell and bulk RNA-seq analyses to assess the impact of αsyn-O treatment on cell-subtype proportions, gene expression, and splicing. We observed a significant increase in the proportion of AC1 (inflammatory) relative to AC2 (neuro-protective) astrocytes in αsyn-O treated as compared to cocultures basally (Fig. 3E and table S4). We noted that there was no significant difference observed in the cell-type proportions of AC1 and AC2 in astrocyte-only cultures on αsyn-O treatment (table S4). Differential gene expression analysis following correction for changes in predicted cell-type proportions, identified 774 genes (3.46%, FDR < 5% and at least twofold change in expression) with 509 up-regulated and 265 down-regulated in the αsyn-O treated as compared to the cocultures basally (table S5). Similar to the findings on αsyn-O–treated astrocytes only, the up-regulated genes with at least twofold change in expression were highly enriched for immune response terms (Fig. 3F and table S10). As observed in the astrocyte-only cultures, the terms highlighted were associated with viral infections, such as “defense response to virus” and “type 1 interferon signaling pathway.” Furthermore, we noted that gene enrichment ratios for immune-related Gene Ontology (GO) terms were consistently higher in αsyn-O–treated cocultures compared to astrocyte-only cultures, suggesting a more prominent inflammatory response to oligomer treatment in cocultures (Fig. 3G and table S11).

Again, analysis of differential splicing identified distinct biological processes that were related to structural organization of cells and physical cell interactions, in contrast to gene level expression signals. We identified 502 differentially spliced intron clusters in 414 genes (FDR < 0.05, |∆PSI| ≥ 0.1), with the genes enriched for terms relating to junction assembly and synapse (table S7). Last, we assessed differentially expressed and differentially spliced genes for evidence of enrichment for genes genetically associated with either Mendelian forms of early onset PD and Parkinsonism, or complex PD (*42*, *43*). While we did not see any enrichment of Mendelian genes in astrocyte-only cultures, there was significant enrichment among all genes that were differentially expressed or differentially spliced (table S12) in astro-neuronal cultures. Overall, this suggests that this model is related to PD pathogenesis and uncovers pathways related to PD causation.

### Oligomer treatment triggers *ADAR* expression and a change in isoform use

**Fig. 4.**
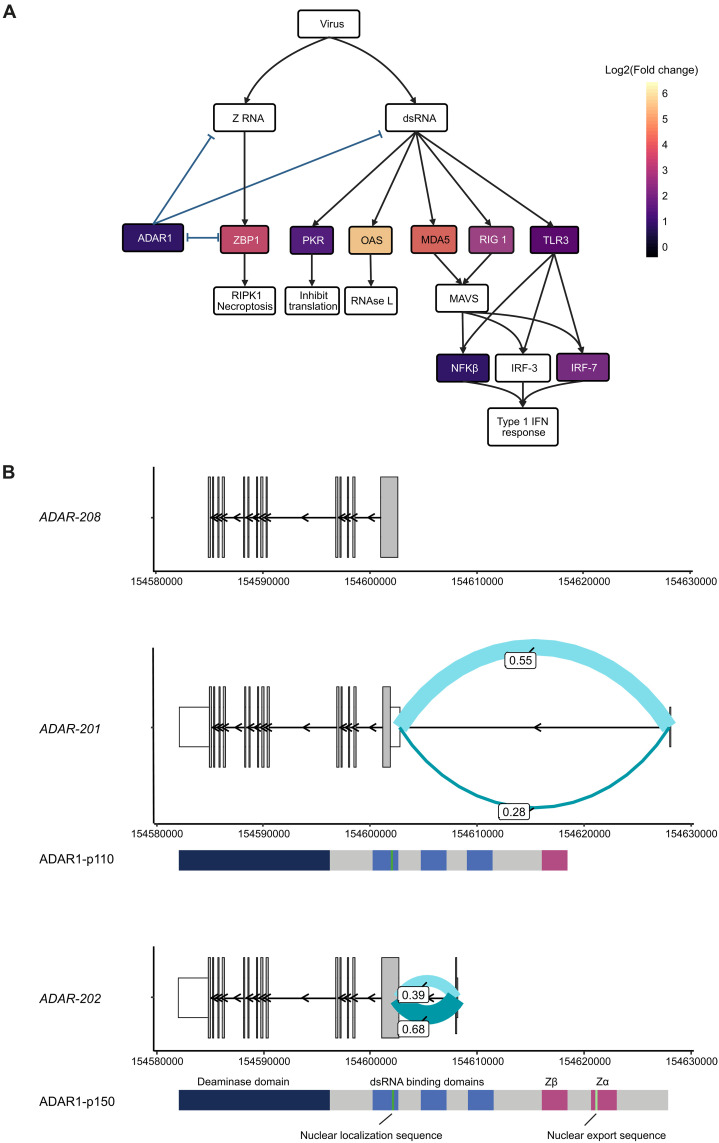
Type 1 IFN response leads to activation of *ADAR.* (**A**) Model summarizing the role of *ADAR* in regulating the innate immune response to dsRNA with the log_2_(Fold change) of the genes that are significantly differentially expressed (at FDR < 5%) in the astrocytes on αsyn-O treatment. Model created with BioRender (https://biorender.com/). (**B**) Plot showing the significantly differentially spliced junctions in *ADAR* in the astrocytes on αsyn-O treatment, with the transcripts and protein isoforms. (Coculture, fig. S5, A and B).

αsyn-O treatment of both astrocyte-only and astro-neuronal cultures resulted in the activation of pathways most commonly associated with responses to viruses. In both cases, we noted significant increases in the expression of genes such as *MDA5*, *RIG1*, and *TLR3* that can sense viral RNA [double-stranded RNA (dsRNA) or Z-RNA] (*44*–*46*) and activate the release of IFN and cytokines (Fig. 4A and fig. S5B). This in turn is known to trigger the up-regulation of a range of genes, including *OAS1*, *PKR*, and *ZBP1* to degrade viral RNA, inhibit translation, and drive necroptosis, respectively, and this up-regulation was identified in αsyn-O–treated cultures (Fig. 4A and fig. S5B).

However, these processes also undergo negative regulation by ADAR, an enzyme that deaminases adenosines on dsRNA to inosines (*47*). This conversion reduces the activation of dsRNA sensors by disrupting RNA self-complementarity and so favoring the formation of single-stranded RNA forms, as well as through other direct and indirect modulation of proinflammatory pathways (*48*–*51*). It is known that biallelic pathogenic variants in ADAR that reduce its editing activity result in excessive release of interferons (IFNs) and tissue damage, and present as Aicardi Goutieres syndrome (*52*). With this in mind, we noted that αsyn-O treatment of both astrocyte and astro-neuronal cultures resulted in significant increases in *ADAR* expression (Fig. 4A and fig. S5C) of 1.86- and 1.82-fold in the cultures, respectively.

Furthermore, αsyn-O treatment resulted in significant differences in transcript use (Fig. 4B and fig. S5A) as detected through splicing analyses. More specifically, we noted a decrease in the use of an exon-exon junction specific to *ADAR*-201 (1:154602627-154627854:-) encoding the ADAR p110 isoform, and a relative increase in the usage of an exon-exon junction (1:154602627-154607991:-) specific to *ADAR*-202 encoding the p150 isoform, which is already known to be under the control of an IFN-sensitive promoter (Fig. 4B and fig. S5A). Thus, the functional and transcriptomic analyses of astrocyte-only and astro-neuronal cultures, suggest that αsyn-O treatment triggers the increased expression of *ADAR* and an increase in the use of the cytoplasmic p150 isoform.

### αsyn-O treatment increases A-to-I editing in astrocyte-containing samples

**Fig. 5.**
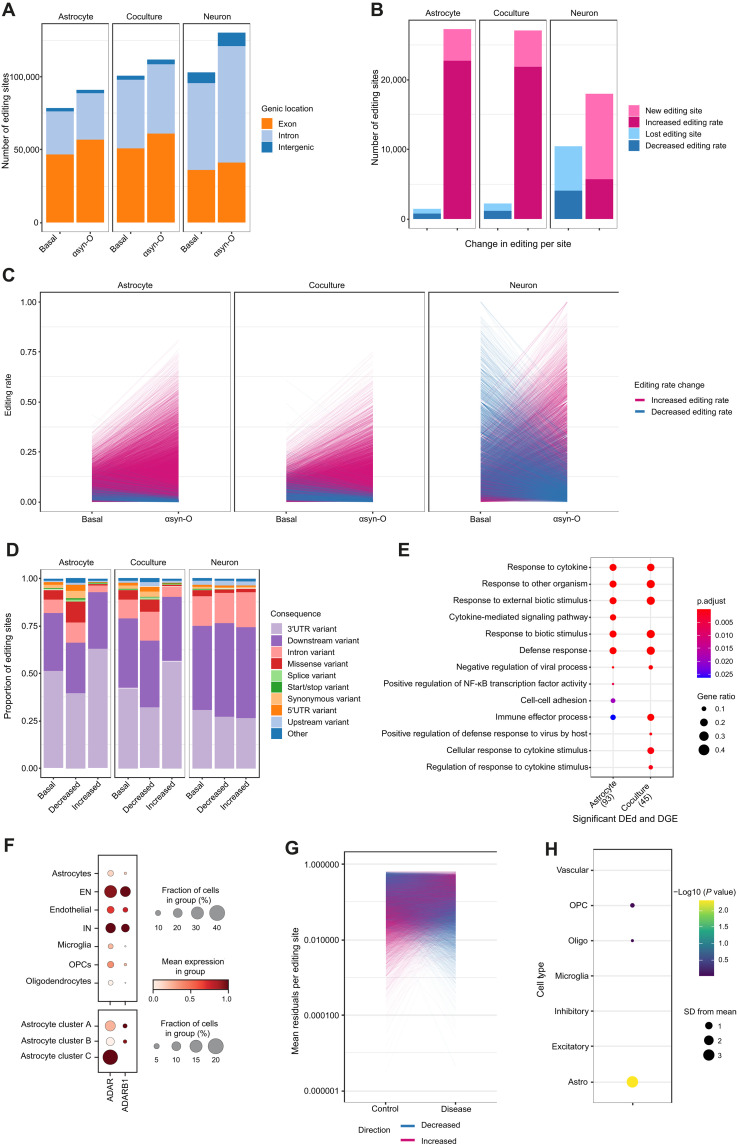
*ADAR* induced in A-to-I editing in astrocytes and cocultures with αsyn-O treatment. (**A**) Number of editing sites in each sample, showing genomic locations. (**B**) Number of differentially edited sites when comparing αsyn-O treated to samples basally in each culture. *X* axis shows the decreased editing (blue, including lost) and increased editing (pink, including new). (**C**) Change in editing rate in basal versus αsyn-O treated samples in each culture. (**D**) Consequences of editing sites at baseline, and in differentially decreased or increased sites across cultures. (**E**) GO terms associated with the significantly differentially edited and differentially expressed (FDR < 5% and FC ≥ 2) in the astrocytes and coculture αsyn-O, treated versus basal. (**F**) Expression of *ADAR* and *ADARB1* in single nuclear postmortem brain RNA. Top, shows expression by cell type, and the bottom shows expression within astrocytic clusters. Astrocyte cluster A is defined by expression of *ADGRV1* and *SLC1A2*; astrocyte cluster B by *GFAP*, *S100B,* and *AQP4*; and astrocyte cluster C by *VIM*, *SOX9*, and *FOS*. (**G**) Mean residuals per editing site, after correction for covariates, in controls and diseased PD postmortem brain samples. (**H**) Expression weighted cell-type enrichment analysis of genes with 50 greatest increases in mean editing rate. Abbreviations: EN, excitatory neurons. IN, inhibitory neurons. OPC, oligodendrocyte progenitor cells.

We postulated that changes in *ADAR* expression and its isoform use would result in both an increase in A-to-I editing rate and a change in the distribution of A-to-I editing sites. The latter would be expected as a consequence of the different properties of ADAR’s two major isoforms, with the p110 exclusively found in the nucleus and the p150 being largely cytoplasmic (*53*). To investigate this, we used the high-depth bulk RNA-seq data we generated across all cultures to identify editing sites, in each case comparing to the reference genome. Focusing on basal conditions, we detected 78,547 editing sites in astrocyte-only cultures, 103,340 sites in the neuron-only cultures, and 100,740 sites in the astro-neuronal cultures (Fig. 5A). Consistent with the known higher levels of editing in neurons (*54*), we found that the median baseline editing rate at a given site was higher in neuron-only (0.208) than in astrocyte-containing cultures (0.054 in astrocyte-only and 0.056 in astro-neuronal cultures). Given that astro-neuronal cultures were composed of ~50% neurons, our findings suggest that in the presence of astrocytes, editing rates in neurons are lower.

This analysis also revealed differences in the distribution of editing sites across cell cultures. In astrocyte-only cultures, 50.3 to 59.5% of editing sites were in exonic regions, with the majority of these located in the 3′ untranslated region (3′UTR) (49.6 and 40.9% of editing sites in astrocyte-only and astro-neuronal cultures, respectively; Fig. 5D). By contrast, in the neuron-only cultures, we found that just 25.4% of sites were located in the 3′UTR. Last, consistent with the known molecular function of *ADAR*, we found that irrespective of genic location or culture type, the majority of editing sites (83.6 to 86.6%) were located within repeat regions, of which the vast majority were in Alu regions (93.2 to 94.5%) (fig. S6C).

As predicted, αsyn-O treatment generated an increase in the number of editing sites in all cultures (15.8% in astrocyte-only, 26.2% in neuron-only, and 11.1% in astro-neuronal cultures), which was highly significant (chi-squared *P* value < 2 × 10^−16^ for increased exonic proportion in all cases). Similarly, measurement of editing rates at each site demonstrated both a marked increase in the number of previously unidentified editing sites and editing rate in astrocyte and astro-neuronal cultures, but not neuron-only cultures (Fig. 5B). In contrast, across all cultures, relatively few editing sites were lost or had a decrease in editing rate.

Furthermore, consistent with a change in transcript usage, αsyn-O treatment was associated with a change in the distribution of editing sites. In astrocyte-containing samples, the sites with increased editing rates were significantly more likely to be in 3′UTRs than sites with decreased editing (*P* value < 2 × 10^−16^ in both astrocyte-only and astro-neuronal cultures), while in neuronal monocultures, the proportion of sites in 3′UTRs did not change significantly (*P* value = 0.17). A similar pattern was observed when exploring the biotype (as defined by Ensembl VEP 93.5, RRID:SCR_002344) of the transcripts containing a given editing site, with sites identified to have an increase in editing rate in astrocyte-containing cultures being more likely to be located within protein coding transcripts (*P* values < 8.2 × 10^−8^ and 2 × 10^−16^ in astrocyte-only and astro-neuronal cultures, respectively; fig. S6B). Together, these results show that αsyn-O treatment is associated with an increase in the number of editing sites and differential editing rate in astrocyte-containing cultures.

Noting that RNA editing can influence gene expression through effects on mRNA stability, we explored the relationship between transcript editing, gene expression, and gene function (*55*). We began by identifying all genes that both contained sites with significant differential editing on exposure of cells to αsyn-O treatment and that had significant differences in gene expression in the same conditions. We found that there was a significant overlap in genes that were differentially edited and differentially expressed in the astrocyte-only and astro-neuronal cultures following αsyn-O treatment (Fisher’s exact test *P* value for astrocytes 8.54 × 10^−4^ and coculture 2.39 × 10^−2^). Focusing on this gene set, namely genes that were both differentially edited and expressed, we found a significant enrichment for the terms linked to viral infection and immune response in both the astrocyte-containing cultures (Fig. 5E and table S13).

### A-to-I RNA editing is increased in postmortem PD brains

While A-to-I editing in the human brain is well-recognized and perhaps best characterized in neurons, the molecular machinery for editing is present in all the major cell types. In humans, A-to-I editing is catalyzed not only by ADAR1 (*ADAR*) but also ADAR2 (*ADARB1*), which primarily edits at conserved sites in the genome (*53*, *55*, *56*). Using publicly available small nuclear RNA-seq data from human brain, we confirmed the expression of *ADAR* in all major cell types including astrocytes (Fig. 5F). In astrocytes, the majority of expression was found in the astrocytic subtype expressing *VIM*, *SOX9*, and *FOS*, with 19.4% of these cells expressing *ADAR*. The mean expression of *ADAR* also appeared to be higher in this subgroup, with *ADAR* among the top 500 most expressed genes. This is in keeping with *VIM*-positive astrocytes being immunoreactive, associating with response to toxins, viruses, and cytokines, activation of surrounding neurons, projections over extended distances in the CNS, and astro-vascular interactions (*57*–*59*).

To explore whether the changes in A-to-I RNA editing seen in vitro were also reflected in PD-affected human brain tissue, we explored RNA editing in a publicly available dataset of five control and seven PD postmortem brain samples (*60*). Using high-depth RNA-seq data generated from the anterior cingulate cortex, and after controlling for covariates, we found that PD brain samples had significantly higher levels of RNA editing than control samples [β 0.011, 95%, *P* < 2.2 × 10^−16^ 95% confidence interval (CI) (0.009, 0.012)]. This association remained significant in a further analysis, which also included the total number of editing sites per sample [β 0.007, 95% CI (0.006, 0.009)]. Next, we assessed the cell-type specificity of the editing response. Focusing on genes with the greatest increase in mean editing rate in PD (top 50 genes), we used expression-weighted cell-type enrichment analysis (*61*) to formally assess the cell-type specificity of this gene set. We found significant enrichments for multiple glial cell types, including oligodendrocyte progenitor cells and oligodendrocytes. However, the cell type with the most significant enrichment was the astrocytes (3.8 SDs increased from the mean, adjusted *P* value 0.0014), suggesting that astrocytes are involved in this process in vivo (Fig. 5G) although not exclusively.

## DISCUSSION

Astrocytes are the most abundant glial cells, supporting neuronal health and CNS immune responses through multiple heterogeneous reactive and proliferative states (*62*). While several triggers for these states are well recognized, it remains less clear how different glial states contribute in the context of diseases associated with proteinopathies. Here, we generated a platform of hiPSC-derived astrocytes to investigate the intersection between protein aggregation as a trigger for astrocytic state switching in disease. We used a serum-free small-molecule approach to generate functionally active astrocytes, demonstrating homeostatic calcium responses, glutamate uptake, and maturation of neuronal function, as well as responses to inflammatory stimulation by canonical microglial triggers [lipopolysaccharide (LPS)]. Adopting single-cell sequencing to characterize the molecular identity of the astrocytes revealed two astrocytic subclasses, protective and inflammatory, recapitulating key astrocytic states seen in the CNS and associated with PD (*13*). Furthermore, the transcriptomic signatures of our iPSC-derived astrocytes correlated highly with iPSC-derived astrocytes generated by other groups (*27*). Our cellular platform may therefore be used to model astrocytic states and substates and uncover the mechanisms that underlie them despite the inherent limitation of their developmental fetal phenotypes (*63*).

The innate immune response recognizes pathogen-associated molecular patterns (PAMPS) and DAMPS via pattern recognition receptors [including TLRs and retinoic acid–inducible gene I (RIG-I)–like receptors] (*64*–*66*). The role of α-syn as a PAMP/DAMP is not well understood. It is currently known that α-syn may be transferred from neurons to astrocytes in vitro (*67*), or from astrocytes to neurons (*68*), and several mechanisms of astrocytic uptake and transfer have been proposed, including endocytosis (*69*) and tunneling nanotubes (*70*), or via TLR2 activation enhancing the uptake of fibrils (*71*). We previously demonstrated that physiological concentrations of oligomers provoke an immunological response that is largely TLR4 dependent and that glial TLR4-Myd88 signaling in the substantia nigra may be causative in PD pathogenesis (*18*).

Here, we used the same α-syn aggregate species to reveal the downstream consequences of recognition by TLRs and the underlying mechanisms of astrocytic reactivity driven by protein aggregation. Our data confirm that oligomers are recognized as DAMPS/PAMPs [driven by genes *TLR3* and *DDX58* (RIG-I)]. Typically, this is followed by activation of key inflammatory transcription factors, nuclear factor κB (NF-κB) and IFN regulatory factors (IRFs) that induce the release of type I IFN. The initial type I IFN release consequently induces the phosphorylation of IRF7 and phosphorylation of signal transducers and activators of transcription 1 (STAT2) and STAT1, which form a complex with IRF9, known as the IFN-stimulated gene factor 3 (ISGF3). Oligomers induce the expression of *NFκB*, *IRF7*, *STAT1*, *STAT2*, and *ZBP1*, reflecting this pathway in the hiPSC astrocytes. Last, the DAMPS/PAMPS trigger the activation of the NLRP3 inflammasome that facilitates the activation of caspase-1 and release of proinflammatory cytokines interleukin-1β (IL-1β), IL-18, and pyroptosis, a sequence also triggered by oligomers (*NLRP3*, *CASP1*, and *IL1B)*. This transcriptomic response was mirrored functionally with the morphological switch to an activated astrocyte, release of inflammatory cytokines, and generation of ROS. Moreover, in this reactive state, the astrocytic supportive function of promoting neuronal activity was lost, and neuronal toxicity was induced. Together, these results suggest a mechanism whereby αsyn-Os can trigger an inflammatory response in astrocytes with resulting glial and neuronal toxicity.

Intracellular dsRNA, evolutionarily a sign of viral infection, can also act as a DAMP, and dsRNA triggers multiple cytoplasmic receptors including ZBP1, MDA5, OAS, and PKR which activate various arms of the innate immune cascade, including NF-κB signaling and the type 1 IFN response. Of the broad changes in gene expression observed in our experimental paradigm, oligomer-induced type 1 IFN changes were accompanied by the activation of antiviral response pathways, with up-regulation of the cytosolic double-stranded nucleic acid (dsNA) sensing machinery. This machinery includes activation of the negative regulator ADAR1, an enzyme that performs RNA editing, breaking the homology of dsRNA, and thus dampening the immune response triggered by cytosolic dsNA and type 1 IFNs (*47*). It is important in responding to cytosolic dsRNA that is endogenous in origin and not secondary to viral infection. From an evolutionary perspective, this process is especially important in humans, where expansion of retrotransposition of noncoding repetitive elements, especially alu repeats, has markedly increased the amount of endogenous dsRNA within cells (*72*). The importance of the immune-dampening effect of ADAR-p150 is demonstrated in loss-of-function mutations, which result in Aicardi Goutieres syndrome, an infantile inflammatory encephalopathy that can also cause striatal necrosis, a brain structure implicated in PD (*73*).

ADAR1 has two major isoforms, with the p110 exclusively found in the nucleus and the p150 being largely cytoplasmic. In the hIPSC astrocytes, we observed promoter switching with the induction of the inflammatory isoform of ADAR1-p150. This resulted in a marked increase in A-to-I RNA editing site number and editing rate per site, most prominently in astrocyte-containing samples. The changes in editing were enriched in 3′UTRs, suggesting that treatment with αsyn-O resulted in a higher proportion of editing activity within the cytoplasm, where ADAR1-p150 is known to localize. Furthermore, among genes that were both differentially edited and expressed, we found a significant enrichment for terms linked to viral infection and immune response in both the astrocyte-containing cultures. Thus, the RNA editing is likely to dampen the inflammatory response to the oligomers in vitro.

Last, we explored the role of RNA editing in PD. Using publicly available high-depth RNA-seq data generated from the anterior cingulate cortex from control and PD-affected individuals, we found that PD brain samples had significantly higher levels of RNA editing than control samples. Having identified astrocyte specificity of the RNA editing response in vitro, we assessed the cell-type specificity of the editing response in vivo. Genes with the most significant editing in PD were enriched in multiple glial cell types, but most significantly in astrocytes, confirming the role of astrocyte RNA editing in vivo.

Our work raises a number of outstanding questions: How do the structural motifs of the oligomers act as danger associated molecular patterns and trigger the IFN and editing response, and when does this process occur in the natural history of PD? Is A-to-I RNA editing a beneficial compensatory response to the inflammatory cascade, or does it exacerbate neurodegeneration in PD? Last, while there is overlap between RNA editing and the genetic risk of PD (*74*), the role of altered RNA editing in PD remains unknown.

The findings here provide insights into the mechanism by which inflammation may be implicated in PD pathogenesis: Specific protein aggregates of α-syn may act as a DAMP to astrocytes, trigger inflammation and IFN-like responses, which in turn triggers antiviral dsRNA responses, leading to activation of RNA editing to dampen proteinopathy induced inflammatory responses. In this work, the disease specific trigger for this mechanism was the β sheet–rich, soluble oligomer of α-syn. However, such mechanisms may also be triggered by viral infections, which are believed to be associated with an increased risk of developing PD (*75*). The identification of dsNA sensing pathways provides a potential convergent mechanism between proteinopathy and viral infections in the pathogenesis of PD.

## MATERIALS AND METHODS

### Aggregation of human recombinant α-syn

Human recombinant α-syn monomeric WT or A53T α-syn was purified from *Escherichia coli* as previously described (*76*). Bacterial endotoxins were removed using a detoxi-gel endotoxin removing Columns (Thermo Fisher Scientific) according to the manufacturer’s instruction. Levels in endotoxin-free α-syn were recorded as 0.006 ng/ml (Stomacher 80 Biomaster, Seward) in α-syn preparations (autoclaved endotoxin-free plastics and endotoxin-free water were used at all stages of preparations). Protein was then aliquoted into separate Eppendorfs into filtered phosphate-buffered saline (PBS) (0.02 μM filter, Whatman), flash-frozen, stored at −80°C, and thawed once before use. αsyn-Os were prepared by first diluting in Dulbecco’s modified Eagle’s medium (DMEM) buffer (DMEM + 1% fetal calf serum + 2 mM l-glutamine). The aggregation mixture was incubated for 26.5 hours at 37°C with constant shaking of 200 rpm (New Brunswick Scientific Innova 43, 25-mm orbital diameter) and centrifuged at 14,200 rpm for 10 min to remove any fibrillar pellets. We took aliquots of α-syn solution directly from an aggregation reaction at different time points and used in these experiments, the time point, at the end of the lag phase (7 to 8 hours). For this time point, we generated and characterized a reproducible prep of oligomers plus monomer (about 1% oligomeric and the rest is monomer). Aggregated α-syn was then stored at 4°C until incubated with cells. Human recombinant α-syn monomer (type 1) and human recombinant beta-synuclein monomer were also purchased from StressMarq (for experiments shown in fig. S3).

### hiPSc culture

hiPSCs were derived from donors who had given signed informed consent as part of the EU IMI–funded program StemBANCC and reprogrammed as described (*77*). Briefly, the Cyto Tune-iPS reprogramming kit (Thermo Fisher Scientific) was used to reprogram fibroblasts through the expression of OCT4, SOX2, KLF4, and c-MYC by four separate Sendai viral vectors. Control 1 (C1) and C2 were derived by StemBANCC, and C3 was purchased from Thermo Fisher Scientific. C4 and C5 were purchased from Applied Stem Cell. hiPSCs were maintained on Geltrex in Essential 8 medium (Thermo Fisher Scientific) and passaged using 0.5 mM EDTA.

No iPSC lines were generated as part of this study. All iPSCs lines were obtained from a number of different sources, commercially, or from repositories (part of the EU IMI–funded program StemBANCC).

### Differentiation of hiPSC into neurons and astrocytes

Differentiation of cortical region–specific astrocytes was performed using a modified protocol based on Gupta *et al.* and Seto-Salvia *et al.* (*78*, *79*). Briefly, as demonstrated in Fig. 1B, hiPSC were differentiated into NPCs using an established protocol (*29*). To derive glial precursor cells (GPCs), NPCs were cultured with dual SMAD inhibition for 25 to 30 days, followed by culturing with the neural induction medium supplemented with human fibroblast growth factor 2 (FGF-2, 20 ng/ml) (*29*). The passage was performed twice per week (1:2 or 1:3) using Accutase (catalog no. A1110501, Thermo Fisher Scientific) by vigorously breaking pellets to remove neuronal cells. Upon the appearance of glial morphology (around day 90 from the neural induction), the GPCs were cultured for 7 days with bone morphogenetic protein 4 (BMP4, 10 ng/ml ) and leukemia inhibitory factor (LIF, 20 ng/ml), which activates the JAK/STAT signaling pathway, refreshing the medium every other day. On the 8th day, BMP4 and LIF were withdrawn, and the GPC were further differentiated for maturation in the neural induction medium without human FGF-2. Three to four more passages are required (1:3 or 1:4) until the complete loss of the precursor property, including proliferation. Generation of astrocytes using an adapted protocol was used to generate data on the effect of monomeric in fig. S3 (A and B) (*28*).

For neurons, at around 35 days of induction, the cells were dissociated into a single cell using accutase and approximately 150,000 cells plated either poly-D-lysine (PDL) and laminin-coated glass bottom 8-well slide chambers (Ibidi/Thistle, catalog no. 80826), Geltrex-coated 8-well Ibidi chambers (catalog no. IB-80826), or 96-well plates (Falcon, catalog no. 353219). The medium was replaced every 4 to 5 days, and the cells were used at 60 to 90 days after induction.

### α-syn treatment

All five astrocyte lines (see table S1) were split on the same day and cultured for 5 days, followed by treatment with 1 μM αsyn-O (equivalent of 10 nM oligomer) the next day. The αsyn-O preparation is generated either in buffer or in media at a high stock concentration of 50 μM, which is then diluted into astrocyte media to the working concentration of 1 μM. Therefore, the αsyn-O comparisons are made to “basal” conditions or “media-only” in which the vehicle is the media. After 24 hours of oligomer treatment, both pellets and media were collected for all assays. Positive controls used for astrocytic activation were performed using either LPS or TIC (TNF-α, 30 ng/ml + IL1a, 3 ng/ml + C1q, 400 ng/ml) for 24 hours (*13*).

### Live-cell imaging

Live-cell imaging was performed using an epi-fluorescence inverted microscope equipped with a charge-coupled device (CCD) camera (Retiga, QImaging) or confocal microscope (Zeiss LSM710 or 880 with an integrated metal detection system). For epi-fluorescence inverted microscope, excitation was provided by a xenon arc lamp with the beam passing through a monochromator (Cairn Research), and emission was reflected through a long-pass filter to a cooled CCD camera and digitized to 12-bit resolution (Digital Pixel Ltd., UK); the data were analyzed using Andor iQ software (Belfast,UK). For confocal microscopes, illumination intensity was limited to 0.1 to 0.2% of laser output to prevent phototoxicity, and the pinhole was set to allow optical slice at approximately 1 to 2 μm. Pre-room temperature warmed Hanks’ balanced salt solution was used as a recording buffer. Three to six fields of view per well and at least three wells per group were used to analyze using ZEN, Volocity 6.3 cellular imaging, or ImageJ software. All experiments were repeated at least three to three times with different inductions.

To measure ROS (mainly superoxide), the cells were washed and 2 μM dihydroethidium (HEt, Thermo Fisher Scientific) was loaded in the recording buffer. The recording was performed using an epi-fluorescence inverted microscope equipped with 20× objective after a quick loading to limit the intracellular accumulation of oxidized product, and the dye was present throughout the imaging. Excitation was set up to 530 nm, and emission recorded above 560 nm was assigned to be for the oxidized form, while excitation at 380 nm and emission collected from 405 to 470 nm were for the reduced form. The ratio of the fluorescence intensity resulting from its oxidized/reduced forms was quantified, and the rate of ROS production was determined by dividing the gradient of the HEt ratio after the application of recombinant α-syn against basal gradient (https://doi.org/10.5281/zenodo.14242993).

For [Ca^2+^]c imaging, Fura-2, AM, which is a ratiometric dye with a high affinity for Ca^2+^ was used. The cytosolic Ca^2+^ as well as the rapid transient kinetics and decay times were assessed (https://zenodo.org/records/10608268; https://doi.org/10.5281/zenodo.14242993). 5u MFura-2 was loaded for 40 min and then washed twice before imaging. The fluorescence measurement was obtained on an epifluorescence inverted microscope equipped with a 20× objective. [Ca^2+^] was monitored in a single cell by obtaining the ratio between the excitation at 340 nm (high Ca^2+^) and 380 nm (low Ca^2+^) for which fluorescence light was reflected through a 515-nm long-pass filter. To trace morphological changes, Fluo4 was used and recorded using confocal microscopy.

Cell death was detected using SYTOX Green(SYTOX, Thermo Fisher Scientific), which is excluded from viable cells but exhibits red fluorescence following a loss of membrane integrity and Hoechst 33342 (Hoechst, Thermo Fisher Scientific), which stains chromatin blue in all cells to count the total number of cells (https://doi.org/10.5281/zenodo.14242993). SYTOX (500 nM) and 10 μM Hoechst were directly added into the dishes, and cells were incubated for 15 min. The fluorescent measurements were using confocal microscopy. Hoechst and propidium iodide were excited by 405 nm with the emission between 405 and 470 nm. SYTOX was excited by a 488-nm laser with the emission between 488 and 516 nm. Percent cell death was quantified by the percent between the number of red fluorescent cells in the total number of Hoechst 33342–expressing cells per image.

### Immunocytochemistry

Cells were fixed in 4% paraformaldehyde and permeabilized with 0.2% Triton X-100. Bovine serum albumin (5%) was used to block nonspecific binding before the cells were incubated with primary antibodies either for 2 hours at room temperature or overnight at 4°C. The next day, the cells were washed three times with PBS and incubated with a secondary antibody for 1 hour at room temperature. The cells were mounted with an antifading medium after three times of wash steps (4′,6-diamidino-2-phenylindole was added in the second wash if required) and let dry overnight.

Primary antibodies used are anti-GFAP antibody (Abcam, ab7260, 1:500) and anti-βIII tubulin antibody (Abcam, ab78078, 1:500). Secondary antibodies used are goat anti-chicken immunoglobulin Y (IgY) H&L (Alexa Fluor 488) (Abcam, ab150169, 1:500) and goat anti-mouse IgG H&L (Alexa Fluor 555) (Abcam, ab150114, 1:500).

### Electrophysiology

Patch-clamp recordings of iPSC-derived neurons were performed using an infrared differential interference contrast imaging system on an Olympus BX51WI upright microscope (Olympus, Japan) coupled with a Multipatch 700B amplifier under the control of pClamp 10.2 software package (Molecular Devices, USA), as described in detail previously (*80*, *81*). For the recordings, a neuronal culture or coculture was plated on glass coverslips, placed in a recording chamber mounted on the microscope stage and constantly perfused with a physiological buffer medium. The perfusion medium contained (in millimolar) 126 NaCl, 2.5 KCl, 2 MgSO_4_,2 CaCl_2_, 26 NaHCO_3_, 1.25 NaH_2_PO4, and 10 d-glucose and was continuously bubbled with 95% O_2_ and 5% CO_2_ (pH 7.4) and maintained at 30° to 33°C. Whole-cell recordings were performed using glass pipettes with a resistance of 3.5 to 6 megohm when filled with the intracellular solution. This solution contained (in millimolar): 126 K-gluconate, 4 KCl, 4 MgCl_2_, 2 BAPTA, 4 Mg-ATP, and 0.4 Na-ATP (pH 7.2, osmolarity ~295 mosmol). In the whole-cell (immediately after membrane breakthrough), iPSC-derived neurons were recorded for the resting membrane potential (Vrest), membrane capacitance (Cm), the membrane time constant (τm), and input resistance (Rin). To induce neuronal firing, a series of sub- and supra-threshold rectangular current pulses were applied with a stepwise-increased stimulus intensity at the Vhold set at −60 to −75 mV. The second protocol tested was a slow-ramp current injection, ramped up with a 100 to 200 pA/s slope. The analysis of the AP waveform was performed for the first AP only to quantify the threshold value, the spike amplitude, overshoot, the spike width (duration at half-maximal amplitude), the rates of depolarization, and repolarization phases, as previously described (https://zenodo.org/records/10608268) (*82*).

### Isolation of single cells

#### Collecting cell pellets for bulk RNA-seq

To collect cell pellets, samples were trypsinized or scraped from the culture surface and placed in a 15-ml conical tube. These tubes were centrifuged at 800*g* in a refrigerated centrifuge for 5 min and the culture media decanted. The pellet was resuspended in 10 ml of chilled PBS per tube by pipetting and then centrifuged again using the above parameters before decanting the PBS. For bulk RNA-seq, the cell pellets were frozen on dry ice and stored at −80°.

#### Collecting cell pellets for single-cell RNA-seq

Samples for single-cell RNA-seq followed the procedure above, although instead of freezing, the pellets were resuspended in 1 ml of PBS, and 100,000 cells were transferred to a 1.5-ml Falcon tube. These were centrifuged at 1000 rpm for 3 min at 4°C before resuspending the cells in 20 μl of chilled DPS. Chilled 100% methanol (180 μl) was added dropwise to the cells while gently vortexing to prevent the cells from clumping before fixing the cells on ice for 15 min.

### Single-cell RNA-seq data generation and processing

Between 2400 and 4000 cells were loaded for each sample into a separate channel of a Chromium Chip G for use in the 10X Chromium Controller. The cells were partitioned into nanoliter-scale gel beads in emulsions (GEMs) and lysed using the 10x Genomics Single-Cell 3′ Chip V3.1 GEM, Library and Gel Bead Kit. cDNA synthesis and library construction were performed as per the manufacturer’s instructions. The RNA was reversed transcribed and amplified using 12 cycles of polymerase chain reaction (PCR). Libraries were prepared from 10 μl of the cDNA and 13 cycles of amplification. Each library was prepared using Single Index Kit T Set A and sequenced on the HiSeq4000 system (Illumina) using 100–base pair (bp) paired-end run at a mean depth of 20 to 50 K reads per cell (EGA study id: EGAS50000000751). Libraries were generated in independent runs for the different samples.

The reads were aligned to the human reference genome (Ensembl release 93, GRCh38) using Cell Ranger v3.0.2 (data and code availability). The analysis was carried out using Seurat v3.0 (*83*, *84*) following Seurat’s standard workflow. Cells expressing fewer than 200 genes were excluded from the subsequent analysis. In addition, we excluded cells with more than 3000 detected genes to remove suspected cell doublets or multiplets. Given that certain cell types, e.g. neurons, naturally express higher levels of mitochondrial genes, we applied a 10% cutoff for the percentage of mitochondrial genes expressed to filter out likely apoptotic cells. Using default parameters of Seurat, data for each sample were log normalized across cells and the 2000 most highly variable genes identified. Using the canonical correlation analysis (*84*) to identify anchors, we integrated the samples using Seurat v3 (*83*, *84*), followed by regression of the effect of cell cycle and scaling of the data. Dimensional reduction was performed using 50 principal components (PCs). We used Clustree v0.4.4 and Seurat’s plot functions to visualize the expression of astrocytic and neuronal marker genes across different cluster resolutions (0.05 to 0.5 in 0.05 increments and 0.5 to 1.0 in 0.1 increments) (*85*). A clustering resolution 0.25 was selected as it was the lowest resolution that explained the heterogeneity in the samples. The differentially expressed genes between the clusters of interest were identified using Seurat’s FindMarkers() and the default “Wilcoxon” test.

### Bulk tissue RNA-seq data generation and processing

Libraries for sequencing were prepared using the Illumina TruSeq Stranded mRNA Library Prep kit by loading 50 ng of total RNA into the initial reaction; fragmentation and PCR steps were undertaken as per the manufacturer’s instructions. Final library concentrations were determined using Qubit 2.0 fluorometer and pooled to a normalized input library. Pools were sequenced using the Illumina NovaSeq 6000 Sequencing system to generate 150-bp paired-end reads with an average read depth of ~137 million paired-end reads per sample (EGA study id: EGAS50000000751).

We performed prealignment quality control using Fastp (v 0.20.0) with default settings, for adapter trimming, read filtering, and base correction (*86*) (Data and code availability). Processed reads were aligned to the GRCh38 human reference genome using 2-pass STAR (v 2.7.0a), with gene annotations from Ensembl v93 (*87*, *88*). Parameters were set to match ENCODE options (https://github.com/RHReynolds/RNAseqProcessing/blob/master/alignment/STARmanual.pdf) except, we only retained uniquely mapped reads and used STAR’s default of a minimum 3-bp overhang required for an annotated spliced alignment. Post-alignment quality metrics were generated using RSeQC (v2.6.4) and MultiQC (v1.8.dev0) (*89*, *90*). We found that an average of 90.4% reads was uniquely mapped.

The processed reads were also quantified with Salmon (v 0.14.1) using the mapping-based mode with a decoy-aware transcriptome based on GRCh38 and Ensembl v93 as the reference (*91*). Salmon’s options correcting for sequence, nonuniform coverage biases (including 5′ or 3′ bias), and GC bias in the data were enabled, and the R package tximport used to transform Salmon transcript-level abundance estimates to gene-level values (*92*). Pipeline source code can be found in https://github.com/RHReynolds/RNAseqProcessing.

### Deconvolution

Cell-type proportions in the bulk tissue RNA-seq samples were estimated using Scaden (v1.1.2) (*36*). Scaden trains on simulated bulk RNA-seq samples, generated from tissue-specific single-cell data, and predicts cell-type proportions in bulk tissue RNA-seq data. The training data were generated using the raw counts and cell types based on the clustering 0.25 from the single-cell data. Thereby, we created 2000 artificial bulk tissue RNA-seq samples by randomly selecting 3000 cells from the total of 8132 cells. Prediction of the cell-type proportions was made using the default parameters and the Scaden developers’ recommendations. Following deconvolution, significant differences in the cell-type proportions between the αsyn-O–treated and basal astrocytes and coculture were investigated using a paired *t* test and multiple comparison correction using Benjamini and Hochberg method, per cell culture. This was only applied to cell types with proportions ≥ 0.01, which included astrocyte clusters 1 and 2 and neuron clusters 1, 2, and 3.

### Differential gene expression analysis

Sources of variation in bulk tissue RNA-seq data were assessed by performing principal component analysis on the gene level expression filtered to include genes expressed in all samples of each cell culture and treatment. We found that cell culture and cell-type proportions were significantly correlated with the first PC (fig. S3B). The individual, age, and sex correlated with PC2. Individual, RIN, and astrocyte cluster 2 correlated with PC3, while culture, individual, age, and neuron cluster 5 were significantly correlated with PC4. The treatment applied to the cell culture correlated with PC5. Accordingly, PC axes 2, 3, and 4 were included as covariates in the model for differential expression and splicing analyses of the bulk-tissue RNA-seq data. Bulk-tissue differential gene expression was examined using DESeq2 (v1.30.1) (*93*), including only genes expressed in all samples within a cell culture and treatment group, collapsing across individuals in a cell culture and treatment group, and controlling for covariates. FDR correction for multiple testing was performed using the Benjamini and Hochberg method, and a cutoff of FDR < 5% was used to consider a gene as significantly differentially expressed.

### Differential splicing analysis

Differential splicing analysis was conducted using Leafcutter (v0.2.9) (*94*). It detects changes in alternative splicing events by constructing clusters of introns that share splice sites and determining the difference in intron usage, measuring differential splicing in terms of the change in the percent spliced in (ΔPSI). Splice junctions outputted by STAR were filtered to remove those with length < 25 nucleotides and regions that overlapped the ENCODE blacklist regions (https://github.com/Boyle-Lab/Blacklist/tree/master/lists) (*95*). The junctions were annotated using junction_annot() from dasper (*96*), classifying them into the following categories, [based on if one end (acceptor or donor) or both ends match the boundary of a known exon]—annotated, novel acceptor, novel donor, novel combination, novel exon skip, unannotated, and ambiguous gene (mapped to more than 1 gene). Those annotated as ambiguous were excluded from this analysis. Leafcutter was run to identify intron clusters by excluding introns of length greater than 1 Mb and those that were supported by <30 junction reads across all the samples or < 0.1% of the total number of junction read counts for the entire cluster. Differentially spliced clusters were identified pairwise, in treated versus untreated astrocytes and coculture samples, using leafcutter’s default parameters and controlling for covariates as identified by the gene level expression. A total of 40,892 and 44,390 clusters (that lie in a single gene) were successfully tested for differential splicing in the astrocytes treated versus untreated and coculture treated versus untreated respectively. Multiple testing corrections via FDR estimation were done, with an intron cluster and its overlapping gene considered differentially spliced at FDR < 0.05 and if the intron cluster contained at least one intron with an absolute delta percent spliced-in value (|∆PSI|) ≥ 0.1. Functional enrichment analysis was performed using clusterProfiler (v3.18.1) (*97*). GO overrepresentation analyses were run and comparisons between genelists made using compareCluster(). FDR correction for multiple testing was done and terms with FDR < 0.05 were considered significant. We analyzed differentially expressed genes at FDR < 5% and with at least ≥ twofold change in expression and differentially spliced genes at FDR < 5% and |∆PSI| ≥ 0.1 .

ADAR’s differentially spliced transcripts were linked to the protein isoforms by first identifying the transcripts the differentially spliced junctions overlapped with. The junctions overlapped with transcripts ADAR-201 and ADAR-202. The number of amino acids in these transcripts matched to P55265-5 (synonym p110) and P55265-1 (synonym p150) in UniProt (*98*) respectively. We further ran multiple sequence alignment of the amino acid sequences from UniProt and Ensembl (obtained from the in silico translated mRNA is translated) for each of the isoforms, that was a match. ADAR’s differentially spliced junctions were visualized with ADAR’s protein coding transcript structures using ggtranscript (*99*).

### A-to-I editing

RNA editing analysis was undertaken with JACUSA2 v2.0.2 (https://github.com/dieterich-lab/JACUSA2), leveraging GNU parallel v20230722 (https://gnu.org/software/parallel/) (*100*–*102*). This uses a drichilet multinomial distribution to ascertain whether transcripts are edited at a genomic site in two modes: In “detect” mode, it compares transcripts against a reference genome identifying editing in individual samples; in “differential” mode, it will compare two samples against each other, looking at sites that are differentially edited in one sample compared to another. Noting that transcripts with increased editing might not be successfully mapped during the alignment step, multi-sample 2-pass mapping with STAR v2.7.9a (https://code.google.com/archive/p/rna-star/) was rerun, allowing a more generous mismatch rate of 16 bp per 100 (*87*). This did not increase the rate of multimapping during alignment. PCR duplicates were identified by samtools v1.13 markdup (https://htslib.org) (*103*). A-to-I editing was assessed in properly paired, nonduplicate reads, with settings to exclude any potential editing sites near the start and end of reads, indel positions and splice sites, as well as sites within homopolymer runs of more than seven bases. An editing site was considered significant if it had an absolute *z* score greater than 1.96 (|*z*| ≥ 1.96). Replicates for each sample group were input to JACUSA2 detect to output a list of editing sites for each of the six groups: astrocytes untreated, astrocytes treated, coculture untreated, coculture treated, neuron untreated, and neuron treated. Differences in editing sites and rates were explored using R (v 4.2, www.r-project.org). JACUSA2 differential was used to ascertain those sites that were differentially edited in treated samples of each cell line, compared to untreated. Edits were annotated with Ensembl’s variant effect predictor (VEP, v93.5, https://ensembl.org/info/docs/tools/vep), filtering duplicate results by consequence, and biotypes of interest (*104*). Where results were not derived with Ensembl VEP, a manual annotation was undertaken using the Ensembl GTF file, deriving genic location and biotype. Editing sites were also annotated with repeat motifs downloaded from RepeatMasker v4.0.5 (http://repeatmasker.org/) (*105*). Functional enrichment of the differentially edited genes (FDR < 5%) that were also differentially expressed (FDR < 5% and at least twofold change in expression) was examined using clusterprofiler (v3.18.1) (*97*).

### Postmortem brain-editing analysis

Postmortem human brain samples were sourced from publicly available data, including bulk and single-nuclear RNA-seq data from five control and seven PD anterior cingulate cortex samples from donors with Braak stage 5-6 disease (https://ega-archive.org/ study ID: EGAS00001005305; https://ncbi.nlm.nih.gov/geo/ accession ID: GSE178146) (*60*). Single-nuclear gene expression was explored in python 3.9 (www.python.org) using the pl.dotplot function from scanpy 1.7.2 (*106*). As with the cellular models, bulk transcriptomic samples were passed through the editing pipeline including trimming with fastp, alignment with STAR allowing 16 base mismatches per 100, and then identification of editing sites using JACUSA2 as above. Using R 4.2.0, sites were filtered to include those present in at least two samples per PD and control group and presence in both groups. Sites with editing rate greater than 0 and less than 1 were input into a linear regression as follows: Editing_rate ~ Disease_Group + Sex + RIN. Expression weighted cell-type enrichment (EWCE v1.11.3, nathanskene.github.io/EWCE/index.html) analysis was undertaken using specificity matrices previously derived for this dataset from single nuclear RNA sequencing results (*60*, *61*). The ranked gene list input to EWCE was defined by the genes with the greatest increase in mean editing rate, relative to all the genes where editing was detected including genes with editing rate of 1.
