# Supplementary material for: Astrocytic RNA editing regulates the host immune response to alpha-synuclein
Source: Sci Adv. 2025 Apr 11;11(15):eadp8504. doi: 10.1126/sciadv.adp8504 (PMC11988446; doi:10.1126/sciadv.adp8504)
Supplement: Supplementary file 1 — Figs. S1 to S7 Legends for tables S1 to S14 [file sciadv.adp8504_sm.pdf]

Science Advances
AAAS

Supplementary Materials for

Astrocytic RNA editing regulates the host immune response to alpha-synuclein

Karishma D'Sa *et al.*

Corresponding author: Sonia Gandhi, sonia.gandhi@crick.ac.uk; Mina Ryten, mina.ryten@ucl.ac.uk

Sci. Adv. **11**, eadp8504 (2025)
DOI: 10.1126/sciadv.adp8504

The PDF file includes:

Figs. S1 to S7
Legends for tables S1 to S14

Other Supplementary Material for this manuscript includes the following:

Tables S1 to S14

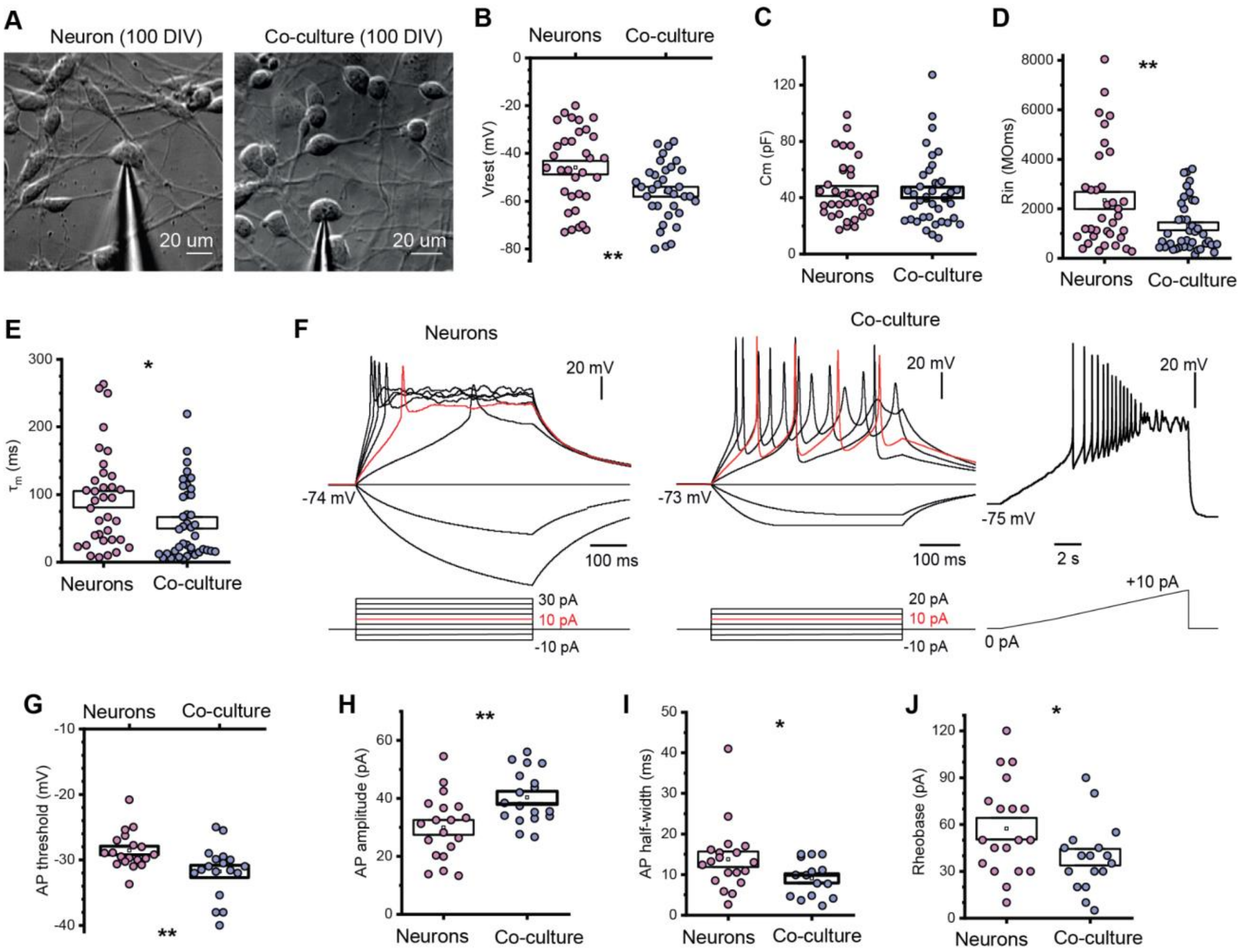

Supp figure 1

Electrophysiological properties of neuronal cells in neuron only and astro-neuronal co-cultures

(A) Transmitted light images of iPSC-derived cells in neuronal culture (left) and astro-neuronal co-culture (right) for whole-cell recordings made from individual neurons using patch pipettes. (B) Statistical summary of the resting membrane potential (Vrest) revealed more hyperpolarised Vrest of iPSC-derived cortical neurons in co-cultures than in the corresponding age-matched cultures. (C - E) Passive membrane properties of iPSC-derived cortical neurons showed similar cell capacity (Cm, Fig 3.C), but different membrane constant (τm, Fig 3.E) and input resistance (Rin, Fig 3.D) between neuronal cultures and co-cultures, indicating a difference in biophysical maturation of the neurons. All data are mean with s.e.m. *$P < 0.05$, **$p < 0.01$ (the two-tailed unpaired t-test). (F) Representative recordings of action potential (AP) firing in an iPSC-derived neuron evoked by a series of rectangular depolarising current pulses of increased intensity (indicated on the bottom) in cultures. Analysis of individual APs across iPSC-derived cortical neurons revealed significant changes in the threshold (G), spike amplitude (H), half-width kinetic parameters (I), and rheobase (J).

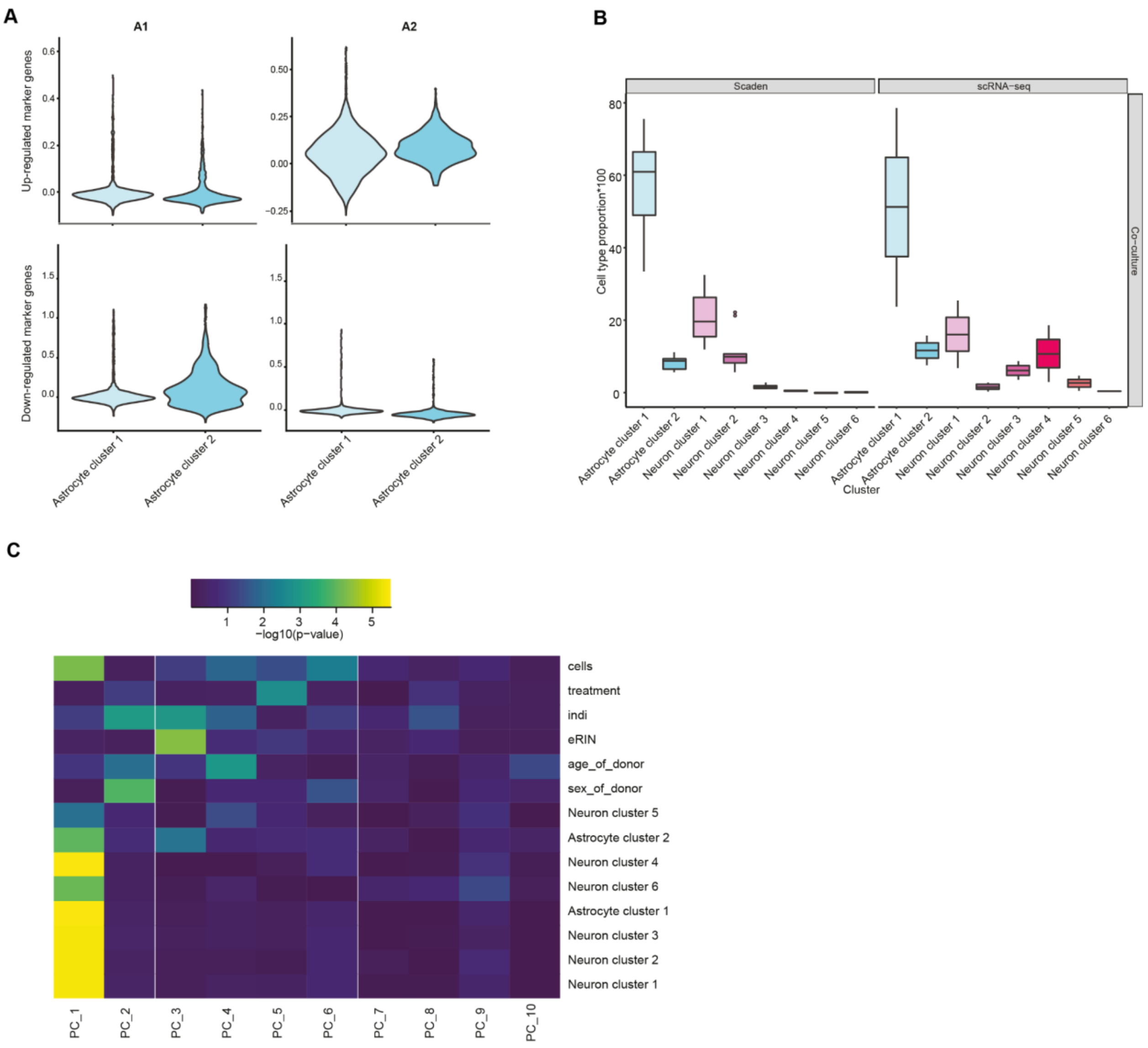

Supp figure 2

Analysis of the single cell and bulk RNA-seq data highlighting astrocyte clusters and cell type proportions

(A) Visualising the module score of the published up and down regulated A1 and A2 marker genes in astrocyte clusters 1 and 2. The module score, generated per cell for a set of genes using the AddModuleScore() in Seurat, is the difference between the average expression of each gene set and random control genes, per cell. (B) Cell type proportions estimated by the single cell data vs that predicted by Scaden's deconvolution (C) Heatmap showing the correlation of the 1st 10 PCs with cell culture, treatment, individual, RIN , sex, age and the cell type proportions of the 8 cell types.

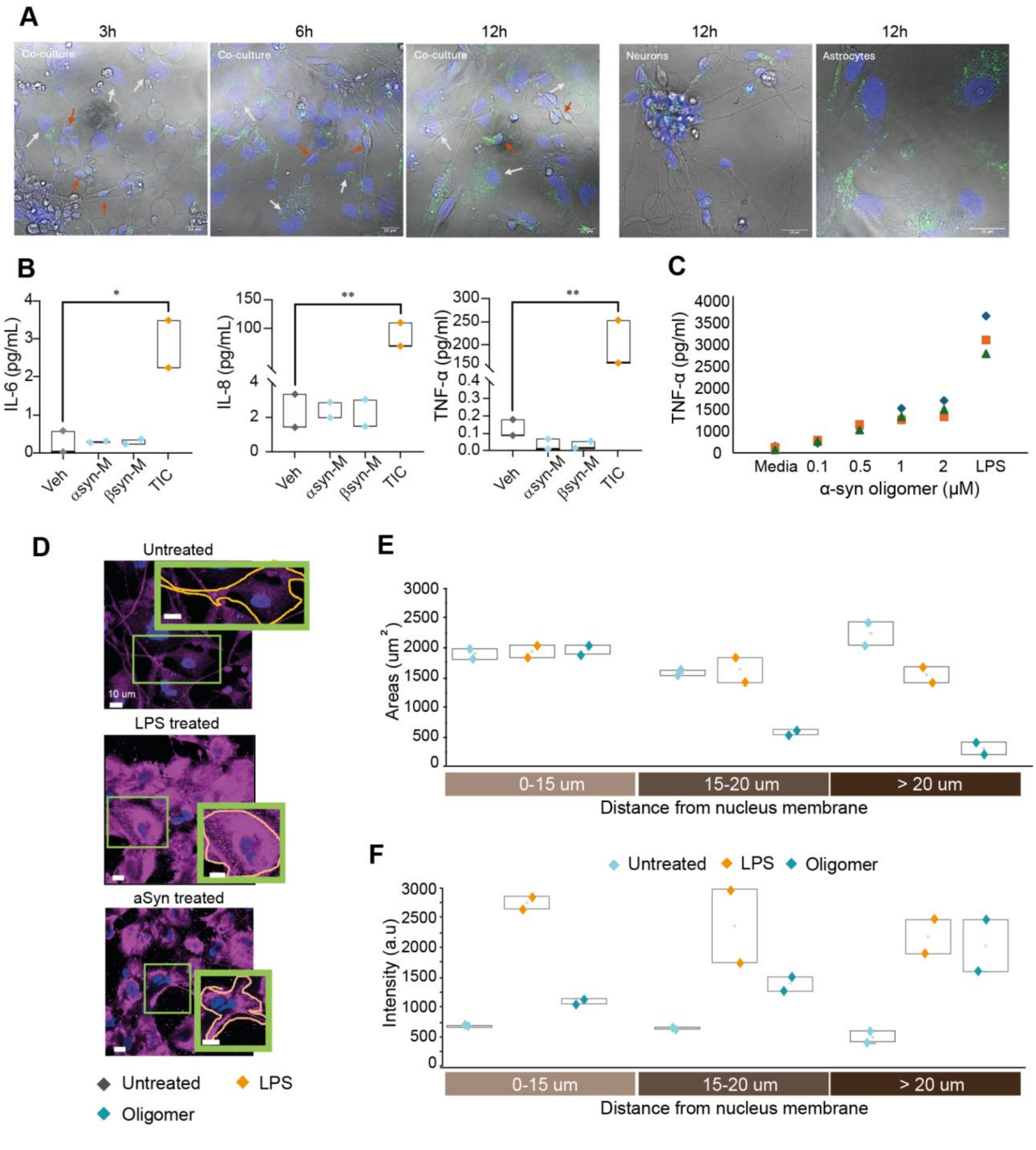

Supp figure 3

Effect of exogenously applied α-syn on astrocytes alone and in co-culture

Astrocyte response to α-syn (A) Mono and co-cultures of astrocytes and neurons treated with fluorescent αsyn monomer 488 (500nM) at different time points (Monocultures treated for 12h; co-cultures treated for 3h, 6h and 12h). (B) Effect of different treatments on cytokine release in cortical astrocytes after a 24h incubation period (IL-6: Veh vs αsyn-M treated $p>0.99$, Veh vs βsyn-M $p>0.99$, Veh vs TIC $p=0.0142$; IL-8: Veh vs αsyn-M treated $p>0.99$, Veh vs βsyn -M $p>0.99$, Veh vs TIC $p=0.0083$; TNFα: Veh vs αsyn-M treated $p>0.99$, Veh vs βsyn-M $p>0.99$, Veh vs TIC $p=0.0073$). Veh, αsyn-M and βsyn-M treated at 1μM; TIC: TNFα: 30 ng/ml + IL1a: 3 ng/ml + C1q: 400 ng/ml. Each data point corresponds to a different donor line. (C) After iPSC-derived cortical astrocytes were treated overnight with αsyn-O or LPS, the media was refreshed and the cells were continued to be cultured for another 24 hours. The

media was then collected to measure TNFα levels using an ELISA kit (R&D Systems).
(D) Representative images of astrocytes immune labeled with GFAP demonstrating change in morphology upon stimulation by LPS or αsyn-O. Assessment of morphology by segmentation of astrocytes followed by (E) assessing GFAP pixel area, according to distance from nuclear membrane (measurement of polarity) and (F) intensity according to distance from nuclear membrane.[38,39]

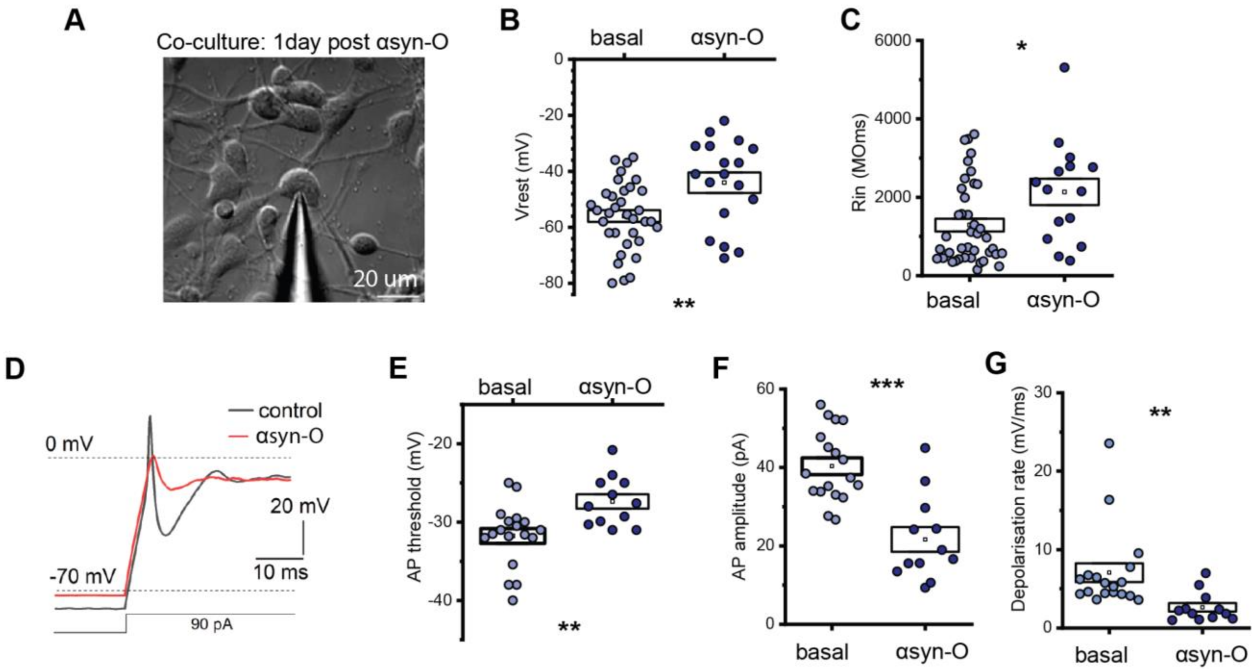

Supp figure 4

Functional effects of αsyn-O treatment in astro-neuronal co-cultures

(A) DIC image of neuron-astrocytic co-cultures for whole-cell recordings of iPSC-derived neurons 1 day after treatment with exogenous mutant α-syn. (B) Analyses of membrane properties revealed a depolarised Vrest in iPSC-derived neurons at 1-day post-treatment with the pathogenic protein. (C) Cortical neurons exhibited an increased Rin (Input resistance) following the treatment compared with the parameters in control (untreated) co-cultures. (D) Representative recordings of action potential spike in a control co-culture (black) and 1 day post-treatment with the pathogenic α-syn (red). Pathological α-syn impaired the shape and kinetics of individual action potential spikes, as measured for the parameters of threshold (E), amplitude (F) and depolarisation rates (G).

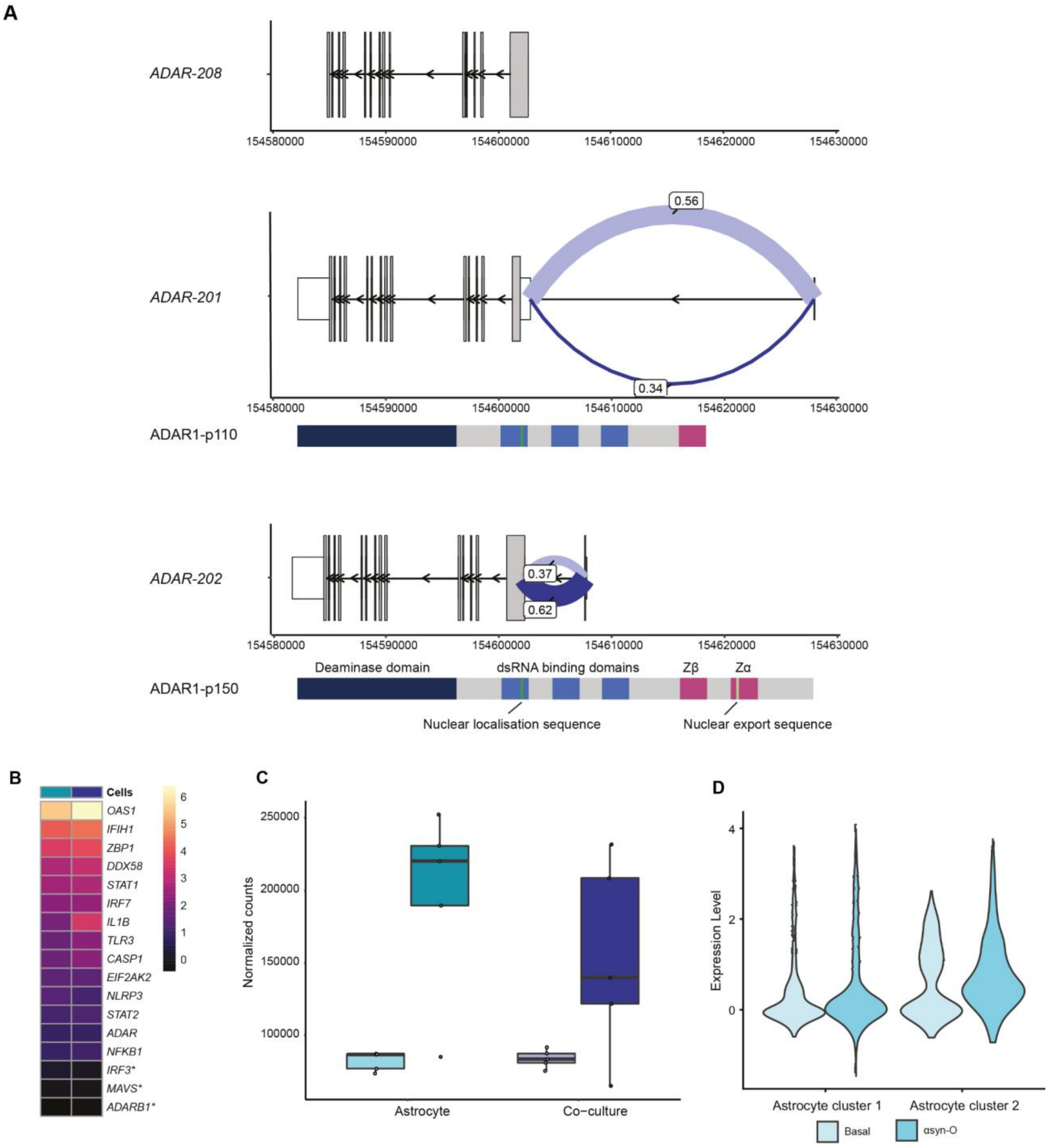

Supp figure 5

Differential gene expression and splicing of *ADAR* in co-culture on treatment with αsyn-O

(A) Splicing in *ADAR* showing alternate isoforms. Junctions are labelled with the junction usage (B) Heatmap showing the $\log_2$FoldChange of the genes, in the innate immune response to dsRNA, (* not significantly differentially expressed at FDR < 5%) in the astrocytes and co-culture on αsyn-O treatment. (C) *ADAR* is differentially expressed in the astrocytes and co-culture on treatment with with α-syn oligomers (D) Expression of *ADAR* in the astrocyte

cluster 1 and 2 in the single cell data

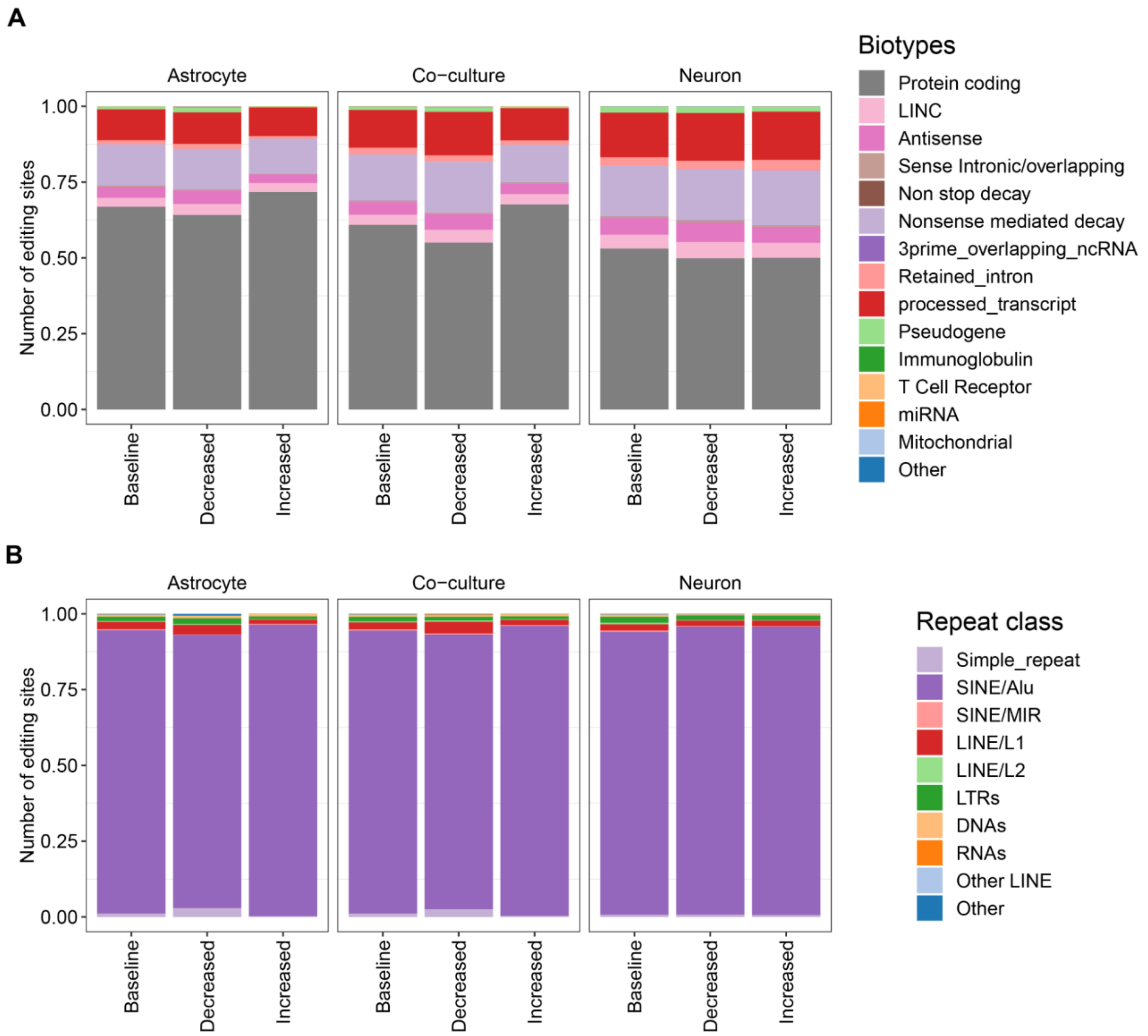

Supp figure 6

Changes in proportion of editing sites with α-synO treatment in astrocytes, co-cultures and neurons showing (A) biotypes annotated with Ensembl Variant Effect Predictor (v93.5). (B) Proportion of repeat regions annotated with RepeatMasker.

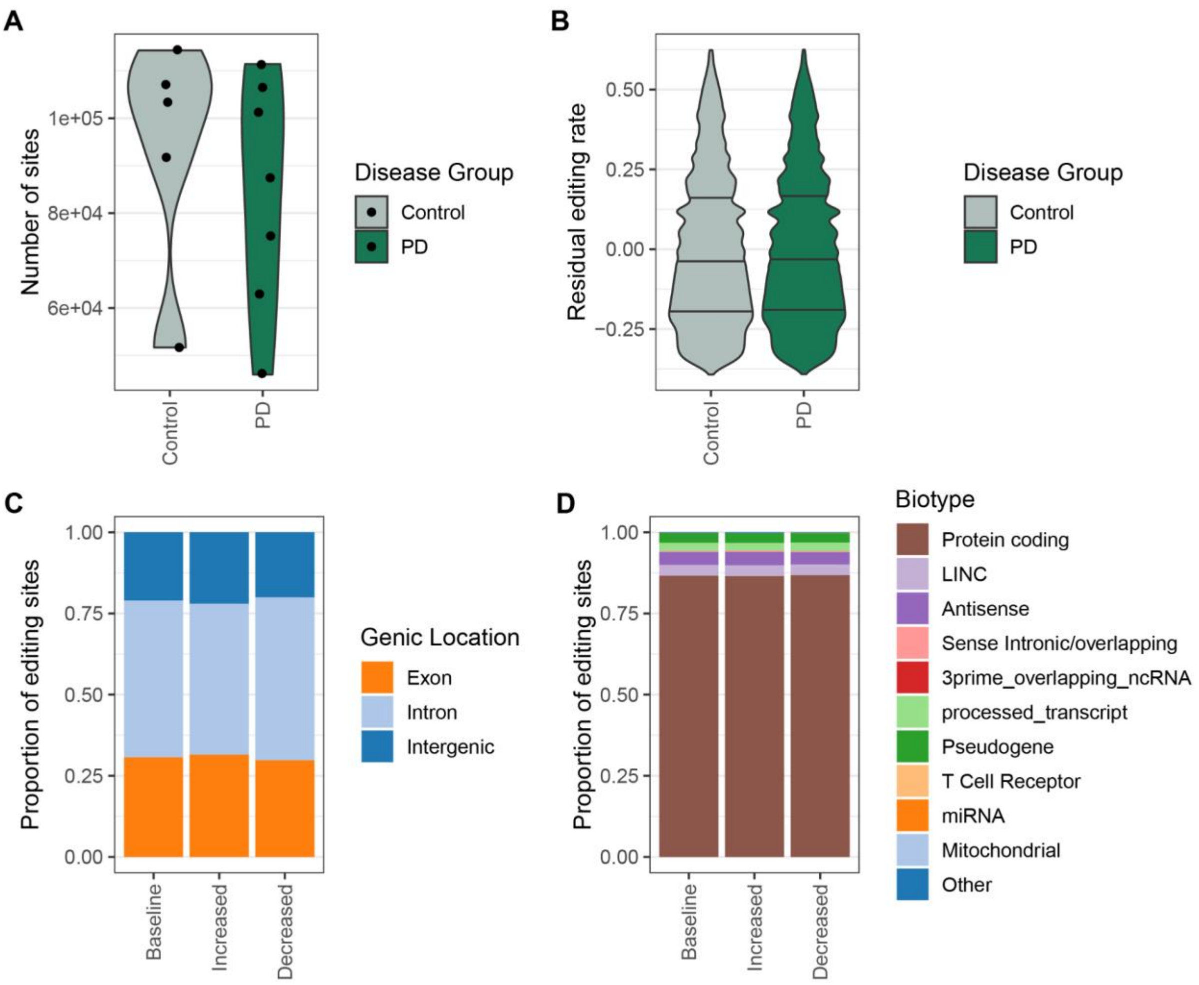

Supp figure 7

A-to-I RNA editing in PD affected human brain. (A) The total number of sites per brain sample. (B) Residual editing rate after correction for covariates. The change in the proportion of sites that have increased or decreased editing, relative to baseline, in genic location (C), and biotype (D).

Supplementary tables

supplementary_tables.xls contains the Supplementary tables 1- 14

**Table**	**Description**
Supp table 1	**Cell lines used in this study.**
Supp table 2	**Genes differentially expressed between the clusters AC1 and AC2, at FDR< 5% and >2 fold change in expression**
Supp table 3	**GO terms associated with the genes up-regulated in Astrocyte clusters 1 and 2 (at FDR< 5% and >2 fold change in expression)**

Supp table 4	**Paired t-test results showing the difference in cell type proportions in the astrocytes and co-culture, between the basal and αsyn-O treated**
Supp table 5	**Differentially expressed genes at FDR < 5% in the astrocytes and co-culture αsyn-O treated vs basal**
Supp table 6	**GO:BP profiles comparing the up and down regulated DEGs in astrocytes αsyn-O treated vs basal(FDR < 5% and at least 2 fold expression)**
Supp table 7	**Differentially spliced genes at FDR < 5% in the astrocytes and co-culture αsyn-O treated vs basal**
Supp table 8	**GO:BP biological process terms - DS genes in astrocytes αsyn-O treated vs basal(Differentially spliced - FDR < 5% & abs(dpsi) >= 0.1)**
Supp table 9	**GO:CC biological process terms - DS genes in astrocytes αsyn-O treated vs basal (Differentially spliced - FDR < 5% & abs(dpsi) >= 0.1)**
Supp table 10	**GO:BP profiles comparing the up and down regulated DEGs in co-culture αsyn-O treated vs basal(FDR < 5% and at least 2 fold expression)**
Supp table 11	**GO:BP profiles comparing the up regulated genes in the astrocytes to that in co-culture on αsyn-O treatment**
Supp table 12	**Table showing the Fisher's Exact Test p-value for the overlap of the differentially expressed and spliced genes (FDR < 5%) in astrocytes and co-culture, genetically associated with Mendelian forms of early onset PD and Parkinsonism, and complex PD (genesets from PanelApp version 1.68 for Parkinson Disease and Complex Parkinsonism ;Nalls et. al, The Lancet 2019) .**
Supp table 13	**GO terms associated with the significantly differentially edited and differentially expressed (FDR < 5% and FC >=2) in the astrocytes and co-culture αsyn-O treated vs basal**
Supp table 14	**Key Resource Table - Protocols, Lab materials, Data, Code, Software availability**
